# A troubling cost—A study of the republican sacrifice in murals

**DOI:** 10.3389/fsoc.2024.1490546

**Published:** 2025-03-05

**Authors:** Fredrika Larsson

**Affiliations:** Department of Military History, Sweden's National Defense University, Stockholm, Sweden

**Keywords:** Northern Ireland, collective memory, trauma, murals, republicanism, IRA, cultural violence

## Abstract

This article explores the representation of the cost of war in mainstream and dissident republican murals in Northern Ireland by examining depictions of bodily sacrifice in three historical rebellions. The study highlights how psychological resilience is valorized alongside physical sacrifice, reinforcing identity hierarchies within republicanism. This challenges assumptions of republican solidarity, revealing competitive dynamics within and between mainstream and dissident factions. Murals as expressions of the republican identity and collective memory show how cultural violence is embedded in the republican collective memory, legitimizing past violence while marginalizing dissenting perspectives. This perpetuation hinders societal healing, as these narratives exclude those who challenge the justification of violence, complicating efforts to address the mental health crisis stemming from the Troubles. The article underscores the critical role of visual culture in shaping collective memory and identity, perpetuating societal hierarchies and cultural violence. It also identifies the potential for reinterpretation of sacrifice, offering a path toward inclusive understandings of the Troubles.

## 1 Introduction

All conflicts result in both physical and psychological wounds and scars. How these wounds are perceived by the public is shaped by the societal context. In some instances, these wounds elicit compassion, while in others, they are met with disgust and a lack of understanding. In certain cases, however, these wounds elevate the individual, marking them as a hero who protected their community or a martyr who sacrificed themselves for it (Jabri, 1996). Within the republican (Irish-Catholic) community in Northern Ireland, veterans of the Troubles (the Northern Irish conflict) are venerated as heroes. Their imprisonment and bodily wounds are perceived as sacrifices that expedite Ireland's reunification in Irish collective memory.

Yet, these veterans are not publicly portrayed as hurt or wounded. Instead, it is suggested that their engagement in the conflict has made them physically and psychologically stronger (Larsson, 2021). However, the Troubles have resulted in significant, unresolved psychological and physical issues within the population (Dybris McQuaid, 2020; Downes et al., 2013). According to Hanna (2012), the Troubles have produced generational trauma, a trauma inherited by successive generations. To cope with this trauma, many individuals resort to self-medication through alcohol and drugs, contributing to a heroin epidemic. Northern Irish society has not emerged from the Troubles unscathed or unharmed (Higgins and McElrath, 2000). While republican veterans are depicted as having been strengthened by the Troubles, the broader society, which witnesses these public displays, remains deeply wounded by the conflict. Sinn Fein, the main republican party, and the broader republican movement are today the leading and largest party in the Stormont. Thus, they bear direct responsibility for mental health aid. In addition to this, Sinn Fein's political capital, as being Provisional IRA's alleged political wing, rests upon their previous involvement in the Troubles (Larsson, 2021). This creates a dichotomy within the republican community regarding how wounds are understood, framed, and their implications for republican identity, thus impacting the perceived cost of the Troubles.

This article will address these issues by exploring how bodily sacrifice is framed within republican collective memory and identity through an analysis of republican murals from 2019 to 2023. This timeframe has been selected due to the varying interpretations of what Brexit signifies for republicans. Both dissident and mainstream republicans agree on the legitimacy of past violence but differ regarding the necessity of sacrifice today (Whiting, 2016, 2015). Dissident republicans, who oppose the peace process and power-sharing, view Brexit as a signal to resume the armed struggle against the British government, thereby justifying the continuation of bodily sacrifice. In contrast, mainstream republicans, who participate in power-sharing and the peace process, argue that bodily sacrifices are no longer necessary as the armed struggle has transitioned into a new phase of cooperation and politics. This group is represented by PIRA and Sinn Féin (Mitchell, 2015). Sinn Féin presents itself as a modern political movement aiming for mainstream acceptance and reconciliation with the past. Simultaneously, Sinn Fein continues to justify the violence during the Troubles internally as necessary for achieving its current political status. Dissidents see Brexit as an opportunity to rebel against the British, whereas the mainstream does not share this view. Republican history is often seen as a series of rebellions—moments when the Irish took up arms to achieve independence. Although each rebellion has failed, republicanism, in this view, is perpetually resurrected through the sacrifice of rebels (Beiner, 2007). This study will examine the portrayal of bodily sacrifice in relation to three rebellions—the Easter Rising, the Hunger Strikes, and the Troubles^1^—within both mainstream and dissident republican murals, to explore the representations of the cost of war on Northern Irish society.

### 1.1 The Northern Irish mural

In Northern Ireland, one of the cultural expressions that articulate the collective identities is the mural. Murals teach the collective identity to future generations. Hence, the republican cost of war has been routinely projected in the republican areas since 1981.^2^ Murals also illuminate the present through the lens of the past, as they are continuously repainted to reflect current circumstances. Republicans' counterparts, the loyalists (British-Protestants), similarly paint murals. In both republican and loyalist areas, murals transform these spaces into exclusive zones where the opposing group is not welcome (Hackett and Rolston, 2009; Jarman, 1997). The thematic content of the murals acts as invisible barriers that exclude the other side and provide a sense of security for the residents (Larsson, 2021). This tradition is almost exclusively associated with the working class, and the republican and loyalist areas are among the most impoverished in Great Britain. The role of murals in expressing, manifesting, and teaching what constitutes a hero, and the cost of war is further complicated by the fact that they are traditionally almost exclusively painted by republican and loyalist paramilitary groups (Goalwin, 2013). Murals have served as a means for these groups to assert their control over the area. Northern Irish paramilitary groups and the communities they inhabit have a “see-saw relationship” (Knox, 2002), where the group simultaneously protects the community and subjects it to violence.

The connection between murals and paramilitarism contributes to the reluctance to analyze murals critically. Moreover, murals are often dismissed as sectarian propaganda or biased interpretations of the events of the Troubles (Vannais, 2001). It is essential to emphasize that republican and loyalist identities are fundamentally opposed; each view itself as a historical victim of the other. As a result, what may seem like an innocuous historical reference to one group can be perceived as sectarian and offensive to the other (Brown and MacGinty, 2003; Dawson, 2010). However, for the Self, the messages conveyed by the murals represent their identity, history, and future. In this article, the Self refers to the community that the mural resides in and represent.

Today, both republican and loyalist murals exist in what Bill Rolston describes as a “mixed economy,” where murals promoting peace and reconciliation stand alongside those featuring paramilitary symbolism (Rolston, 2010). This economy has allowed some republican and loyalist communities to explore their identities outside paramilitary symbolism but in some areas the paramilitary control prevails. In this mixed economy, there is ongoing disagreement about the selection of themes and their visual representation. Controversies often arise over how certain symbols and images from the Troubles are used (Larsson, 2021). One of the primary concerns is the portrayal of the volunteer. Both republicans and loyalists have depicted themselves as “masked men”—masked, armed men (Rolston, 2012). For the community that produces the mural, this symbol represents anonymity and ongoing resistance as well as protection against their enemies, but for the opposing side, it signifies sectarian violence (Santino, 2004). While the masked man is less common in republican areas than in loyalist ones, he is still used in dissident muralism. As the author has shown, for dissidents, it serves today as a reminder to mainstream republicans of their past image, thus challenging their political power by holding up a mirror between the dissident violence and the mainstream violence of the Troubles (Larsson, 2021).

Given their connection to the present and their role in teaching and manifesting collective memories, murals offer a critical gateway for understanding and analyzing Northern Ireland. They represent the complex fabric of violence, trauma, class, and memory that characterizes contemporary Northern Ireland.

### 1.2 The Irish collective memory and identity

Violence has historically played, and continues to play, a central role in Irish identity (Elliott, 2001; Kennedy, 2016). According to English, events such as the Easter Rising (1916), the War of Independence (1919–1921), and the Civil War (1922–1923) have embedded violence and revolution within the fabric of Irish identity and what eventually became the Irish Free State (English, 2003, 2007). The Easter Rising is widely regarded as the birth of the modern Irish state. However, the brutality of the subsequent Irish Civil War has cast a shadow over the Rising, making it difficult for the Irish society to find a shared interpretation of the event (Fitzpatrick, 2016; Foster, 2014). Elliott argues that this hesitation has allowed Northern Irish republicans to claim ownership of the memory of the Easter Rising, positioning themselves as the heirs of the Risers. The republican violence during the Troubles has further complicated this narrative in Ireland, enabling Northern Irish republicans to portray their opponents as traitors to the martyrs of the Easter Rising (Elliott, 2001).

In Irish collective memory, death is often given more significance than life, as the martyr's sacrifice is seen as rejuvenating the community. Beiner describes this as a “triumphalist trauma,” where the future is believed to depend on sacrifice, which will ultimately reverse past failures and lead to the unification of Ireland (Beiner, 2007). Elliott frames this belief as the restoration of “a pure Gaelic Catholic race” to what is rightfully theirs, achieved not through sudden victory, but through endurance and sacrifice, particularly the ultimate blood sacrifice of “dying for Ireland” (Elliott, 2001). In this context, republicanism is viewed as a force or spirit, continuously reborn through each republican sacrifice, ultimately leading to Ireland's unification. This concept can be likened to the Holy Spirit (Fierke, 2012; Spencer, 2015, p. 14). Hence, the idea of sacrifice predates Catholicism, rooted in Celtic myth and society (English, 2007), but the Catholic Church provided the infrastructure for expressing Irish nationalism before partition and independence, thereby shaping collective memory.

The utilization of the sacrifice as a mode of protest and resistance against the powerful derives from the Brehon Laws of Celtic Ireland (Fierke, 2012; Feldman, 1991). It can be observed at different sociocultural levels. An individual republican may sacrifice himself through death, the loss of body parts, or incarceration (Feldman, 1991). On a societal level, failed uprisings against the British are viewed as collective sacrifices, with the “death” of the rebellion paving the way for future uprisings. The Easter Rising exemplifies this, as the execution of its leaders enabled the continuation of the quest for Irish independence (Spencer, 2015; McGrattan and Hopkins, 2017).

In republican collective memory, support often follows sacrifice. For example, the Easter Risers were not widely supported until after their execution, when they were reimagined as martyrs, garnering support for the independence movement. A similar trajectory occurred during the second Hunger Strike in 1981, when the prisoners' public and media-exploited sacrifice transformed them into republican icons and martyrs (Pine, 2011). By employing Celtic symbolism and language, the hunger strikers turned their bodies into weapons, embodying the image of Celtic warrior kings and reinforcing the perception of Ireland as oppressed by a colonial overlord (Sweeney, 1993; Feldman, 1991). Republican murals played a crucial role in this transformation, depicting the hunger strikers as gaunt, suffering men, often drawing stark parallels to Christ's suffering on the Cross. Through this visual representation of suffering, the devout Catholic community could interpret these republicans as men sacrificing for Ireland. However, contemporary republican murals have largely abandoned these representations of suffering, instead favoring a visual language that emphasizes the strength of republicans (Larsson, 2021).

The ability to sacrifice oneself for Ireland is embedded in the Irish collective memory; the spirit of rebellion is believed to awaken in extraordinary circumstances, prompting the Irish to rise against the British. Sacrifice marks the transition of a republican volunteer into a rebel (Feldman, 1991). The republican rebel can be visually and culturally represented in two primary ways: as a Celtic warrior king or as a freedom fighter. The warrior king, descending from the ancient Celtic rulers, possesses deep knowledge of poetry and music, embodying the idea of Ireland as an ancient nation striving to reclaim its independence (Jarman, 1992, 1996). The second portrayal is that of the socialist freedom fighter, where republicans align themselves with other global struggles for self-determination. In this depiction, republicans present themselves as progressive and aligned with the oppressed (McGrattan, 2016).

In republican collective memory, violence, sacrifice, and death are seen as the means to achieve a united Ireland. Yet, in the context of the Northern Ireland peace process, where the armed struggle has ceased, these notions hold little place. The act of self-sacrifice is difficult to reconcile with the practice of power-sharing. This tension has created challenges in narrating the future and determining the role of violence, sacrifice, and death within it. Mainstream republicans argue that the armed struggle has evolved, suggesting that new political tools, such as power-sharing, should be employed to facilitate unification. Dissidents, however, argue that the prospect of a united Ireland remains unattainable by the Agreement,^3^ as it upholds the partition. Thus, needing to continue the armed struggle to end partition (Horgan and Morrison, 2011; Larsson, 2021). Mainstream republicans argue that dissident republicans, with their emphasis on the necessity of violence, are trapped in the past and have failed to adapt to the contemporary realities and future possibilities. In contrast, dissident republicans contend that mainstream republicans have abandoned their foundational roots and ideals (Whiting, 2012).

This internal conflict within republicanism is most prominently expressed in debates surrounding the commemoration of volunteers and the justification for the armed struggle (McGlinchey, 2019). Until the late 1980s, mainstream republicans prioritized the national question over issues of equality, democracy, and rights, viewing equality primarily in terms of nationalist rights, with partition standing as its antithesis (Mitchell, 2015). However, this focus shifted following the 1994 ceasefire, when Sinn Féin redefined their narrative. Amongst other political choices, they altered their murals—removing images of masked men and replacing them with smiling volunteers. It could be argued that the smiling volunteers were the coming Sinn Fein politicians themselves; from masked men to politicians (Rolston, 1997; Larsson, 2021). This rebranding led to significant electoral success, allowing mainstream republicans to position themselves as the “true” peacemakers, often at the expense of Protestant communities, while simultaneously claiming stewardship over the republican collective memory (Mitchell, 2015; McBride, 2017).

To further legitimize their policies, Sinn Féin has strategically invoked the legacy and significance of the Hunger Strikers in contemporary political discourse (Hopkins, 2015). Dissident republicans, however, find the mainstream republican appropriation of deceased republican martyrs deeply offensive (Hoey, 2013). They argue that Sinn Féin merely pays lip service to the ideals and memory of the Easter Rising, cynically exploiting the dead as political instruments (McDowell, 2007; Hopkins, 2015).

## 2 Collective memory, visual representations, and visual culture

Collective memory refers to the socio-cultural framework through which groups position themselves in time and space, presenting the present as a consequence of the past, thus forming the foundation of collective identity. This memory comprises selected past events, interpreted and chosen based on current circumstances (Halbwachs and Coser, 1992, p. 60). This article builds on Olick's (2007) notion that collective memory is a form of negotiation within a group, shaping the understanding of the past and subsequently defining the group's current identity. It is important to recognize that individuals can belong to multiple collective memories, merging these frameworks to construct their unique individual identities (Assmann, 2010, p. 37). In these processes, cultural products, expressions, and symbols play a crucial role in both internally and externally transmitting and expressing memory and identity (Huyssen, 1994, p. 3).

Murals, in this context, are understood as visual representations of collective memory. Images possess embedded meanings and are “empty” until cultural meanings are ascribed to them. Visuality infuses these meanings, transforming the object into a visual representation. Murals reflect the meanings within a culture and thus serve as visual representations of the republican collective identity (Rose, 2022, p. 10–16; Barthes, 2016). Murals engage with their surroundings, with their meanings often heightened by proximity to other murals, or in some cases, they engage in symbolic conflict (Jarman, 1995). Collectively, murals form a visual culture that expresses the republican collective memory and identity. Visual culture, in this sense, refers to how images are integral to the ways meanings are expressed, embedded, and produced within a culture, and how these are transmitted to new generations. It provides an analytical lens for understanding this transmission, as images are part of cultural expression (Morgan, 2005, p. 2–9, 27–33). Therefore, the visual culture of murals articulates the republican collective memory through the meanings and narratives they convey.

The visual representation of bodily sacrifice serves as a lens to understand how the cost of war is depicted. As the body is an expressive form of embodiment that serves as the existential basis of culture, self, social institutions, and society (Williams and Bendelow, 1998, p. 208). It is essential to emphasize that the sacrifices made, including experiences of direct violence, incarceration, and the stresses of combat, are real psychological and physical experiences that have profoundly impacted individuals' bodies (Baker, 2020). However, the significance of these bodily experiences is shaped by how they are positioned and framed within the republican collective memory of sacrifice. An example of this is the hunger strikers' use of Gaelic when speaking to their prison wardens, highlighting the body as an active agent that can transform society (Feldman, 1991, p. 177–179; Yuill, 2007). Within the republican collective memory, by sacrificing themselves, individuals' bodies become communal (Fierke, 2012, p. 108–120). Thus, the volunteers' bodies are imbued with meanings derived from collective memory and visually represented in murals, providing both the individual and the community with ways to comprehend their bodily experiences.

The role of bodily sacrifice in republican collective memory can be further understood through the concept of cultural violence, the third dimension of violence that legitimizes direct (physical) and structural (indirect) violence. Cultural violence manifests in the glorification of martial culture, bloodshed, and the fusion of conflict with a group's founding event or myth. It is embedded in the meanings of symbols and cultural expressions, where agents of violence are perceived as heroes or martyrs who protected and sacrificed for the community. Cultural violence frames the world in dichotomous terms—us vs. them—and permeates the culture's normative system (Ferguson et al., 2018, p. 20; Galtung, 1990; Zulaika, 1988, p. 31–35, 294). To the members of the group, this behavior is normalized and does not stand out. In this sense, I interpret cultural violence as the mechanism that casts these men and women as heroes and martyrs within the republican collective memory, due to their sacrifices.

### 2.1 Analyzing murals

The analysis of murals will be conducted using Barthes's (2016) concepts of denotation and connotation. Denotation refers to the literal interpretation of the image, while connotation involves placing the depicted elements within a broader cultural framework. The ability to read visual material both connotatively and denotatively to identify the preferred interpretation depends on the recognition and decoding of symbols within a cultural code. Symbols acquire meaning through representation and interpretation, thus forming systems of representation that are visually expressed as symbols (Hall, 1997). For instance, within the republican collective memory, a phoenix symbolizes the Easter Rising and the notion of republicans being reborn through their sacrifices. When a phoenix is depicted alongside a man wrapped in a blanket, representing a hunger striker, the 1981 Hunger Strike is integrated into the continuous narrative of Irish rebellions (Beiner, 2007).

There is a preferred reading of visual material, often directed by anchoring messages—specific words and concepts that guide the viewer's interpretation. While cultural narratives mediate individual experiences, individuals retain agency in their responses to these representations (Hall, 1997). The choices of color and visual techniques are instrumental in shaping these responses. Analyzing color choices allows me to explore how the sacrifice is conveyed and how republicans communicate the significance of sacrifice in the present. For example, a colorful mural conveys a narrative of victory, while a mural painted in somber colors underpin sadness. Hence, I understand the color choices as setting the tone of the mural's message.

A mural can consist of multiple forms of images; it conveys multiple narratives to different viewers. Each interpretation of a mural is unique, but there is a shared core of meaning attached to the mural. Mainstream and dissident republicans share the system of representations, but they do not agree on the preferred reading of the murals. The phoenix represent resurrection in both views, but they disagree regarding whether there is a need for continued resurrection; the armed struggle as ended or ongoing (McGlinchey, 2019). To differentiate between these imageries, I have chosen to analyze murals that have been signed by dissident groups. As they have been signed by a dissident organization, I can deter that they do not sign off the current political arrangement with power-sharing. In certain instances, the analysis will “flip the meanings” to examine how the narrative is perceived when an individual does not conform to the expectations portrayed in the murals. In these instances, the analysis directs its focus on what it means to not fulfill the ideal presented by the murals. Additionally, I will ask what these imageries mean to dissidents/mainstreams. This approach enables an exploration of differing meanings and understandings within the visual culture and reach the multiple images the mural harbor.

The selected murals have been chosen due to them visually represent the mainstream and dissident republican interpretations of the rebellions (Viggiani, 2016; McGlinchey, 2019). Moreover, I have chosen to depict figures that are veterans of the Troubles: volunteers within the republican paramilitary groups. There are several past and present murals that depict these rebellions and include different actors. The inclusion of different actors participating within the Troubles was a part of the refashioning of the mainstream republican historical narrative. Previously, the republican murals depicted paramilitaries as fighting back against the British (Larsson, 2021; Rolston, 2010). I have based my selection on them referring to the rebellions and sacrifice, placing the organizations within these rebellions, and depicting volunteers' and their bodies. This study will examine how the portrayal of these veterans both influences and is influenced by the surrounding identity. There is an ongoing dialogue between this portrayal and contemporary understandings of Northern Ireland. Given that all Irish individuals have the potential to become rebels, I interpret the portrayal of the volunteer as a canvas upon which the surrounding community projects its aspirations. Additionally, these murals have been chosen due to them being painted during, and after, the turmoil of Brexit since Brexit was to some dissidents a sign to restart the Troubles. Consequently, challenging Sinn Fein in the Stormont of what to do with power-sharing.

## 3 Analysis

### 3.1 Easter rising

#### 3.1.1 Mainstream

**Figure 1:**
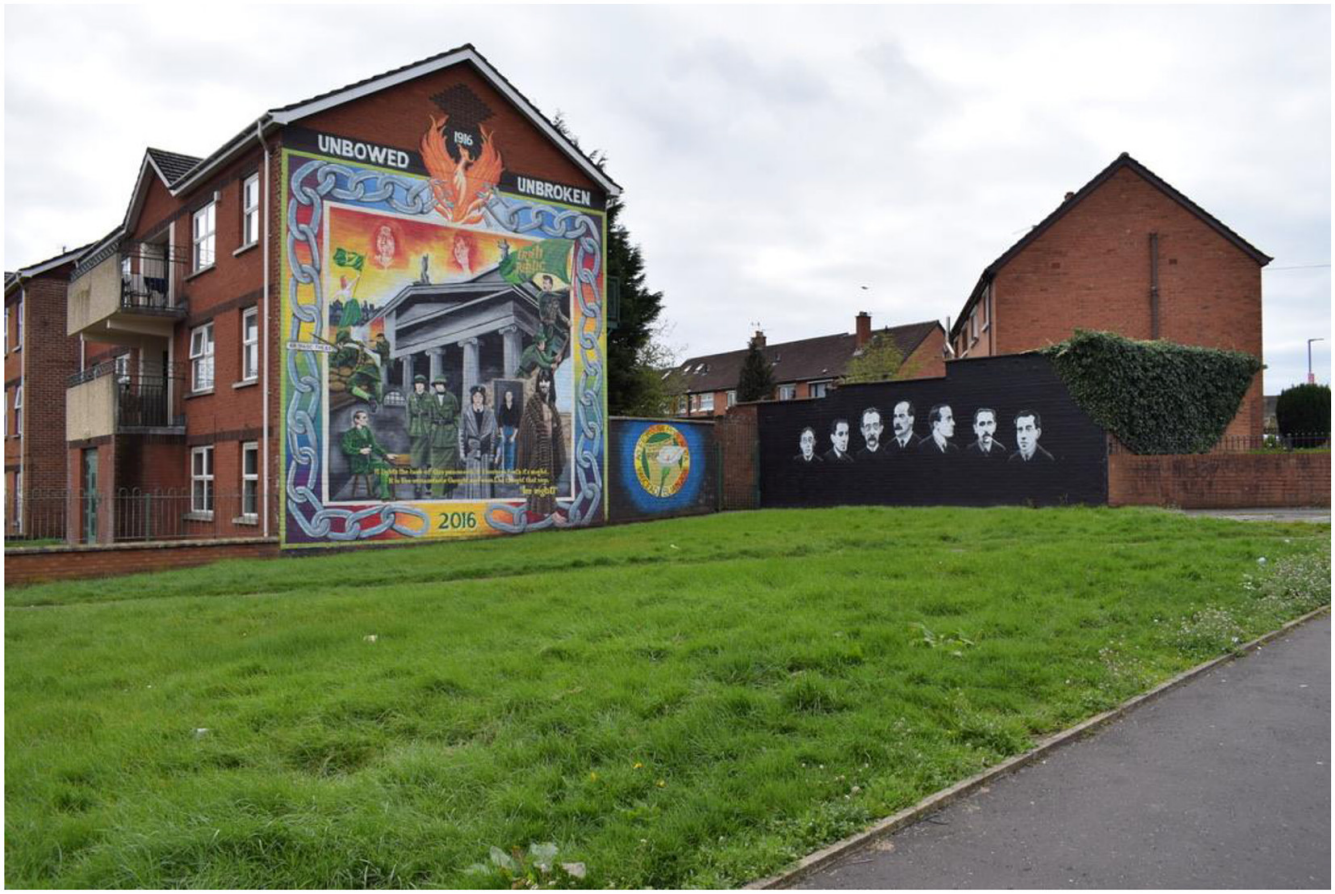
Mainstream republican mural of the Easter Rising in Andersonstown, Belfast.

The Easter Rising holds a central place in the republican collective memory, symbolizing the birth of the modern Irish independence movement. This perspective is vividly depicted in a mainstream republican mural located in Andersonstown, West Belfast (Figure 1). At the heart of the mural is the burning General Post Office (GPO), surrounded by symbols that represent subsequent uprisings born from this pivotal event. These symbols include Padraig Pearse (Easter Rising), a blanket man (the first Hunger Strike), Cuman na mBan (Irish War of Independence), and Mairead Farrell (the Troubles). The imagery of a phoenix rising from the ashes of the burning GPO into the sky, breaking the chains encircling the mural, reinforces the intended message: republicanism will ultimately liberate Ireland. The blanket men participated in the dirty protest, which later became the first and second hunger strike.^4^ Symbolically, the blanket man represents both (Rolston, 2010). The sky, ablaze with republican fire, features the faces of Bobby Sands and Joe McDonnell. McDonnell was a resident of Lenadoon Avenue, not far from this mural, and Bobby Sands is the most prominent hunger striker. He was the first hunger striker during the 1981 hunger strike and was the PIRA commanding officer in prison (English, 2007). The mural's headline, “Unbowed—Unbroken,” is a quote from Bobby Sands that underscores this message: “It lights the dark of this prison cell, it thunders forth its might, it is the undauntable thought, my friend, the thought that says, I'm right.” Adjacent to the mural are the faces of the Easter Risers, rendered in black and white, creating the illusion of ghostly characters offering solemn acknowledgment and approval.

All symbols depicted in this mural represent some form of sacrifice. The connotative reading of the mural makes it a victorious celebration of republicanism, an interpretation grounded in the mural's colors. Yet there is a conspicuous absence of the harsh realities of that sacrifice. Mairead Farrell, for instance, is not shown with the gunshot wounds she sustained when shot by British Secret Service in Gibraltar in 1988. The hunger strikers are depicted as clean and strong, despite the physical degradation and suffering they endured during their slow deaths by starvation. Padraig Pearse is shown calmly awaiting his execution, with no representation of the torment associated with facing a firing squad. The cultural violence embedded in this mural's message suggests that these sacrifices were made effortlessly, with the republican spirit providing the volunteers with peace and protection. Furthermore, this cultural violence implies that the protection afforded by the republican spirit not only shielded the volunteers but also made them stronger than ordinary men, as symbolized by the phoenix's rebirth. For example, the hunger striker is portrayed as healthy, muscular, and robust, rather than as the gaunt, suffering Figure of the 1980s. The mural's headline, “Unbowed—Unbroken,” reinforces this interpretation, implying that the sacrifice did not break the volunteers. This slogan, found throughout Northern Ireland, is often understood to mean that the British could not break them; the republican spirit shielded them from surrendering to their enemies.

Within the republican collective memory, a volunteer becomes a rebel through their sacrifice. In this mural, however, it is suggested that a volunteer becomes a rebel by *surviving* that sacrifice. The distinction lies in the notion that if a volunteer succumbs to the violence and breaks, the republican spirit does not enter and protect them, removing them from the category as a rebel. This creates a dichotomy within the republican ranks: true rebels and those who are deemed inferior since they did not survive their sacrifice: they got psychological problems from their participation in the Troubles. This analysis is made through flipping the meanings of the mural: by studying it through the eyes of someone that is suffering from bad mental health derived from the Troubles. The mural's message is that by not surviving the sacrifice, i.e., participating in direct violence and/or imprisonment means that the British broke them. Those who are broken are portrayed as bowing to their enemies. The cultural violence conveyed in the mural depicts those who are broken mentally and physically as obstacles to the unification of Ireland. This raises questions about how the body is understood within the republican collective memory. In this context, some bodies are deemed unworthy of the struggle due to their perceived weakness and must be left behind on the path to Ireland's unification.

Traditional commemorative murals often depict death and iconized individuals. However, this analysis has shown that embedded in this line of commemoration lies the heart of cultural violence: seemingly mundane cultural expressions that display and express differentiation. In this example, the mural displays and expresses exclusion from the future united Ireland and the republican, in extension, Irish identity.

#### 3.1.2 Dissident

**Figure 2:**
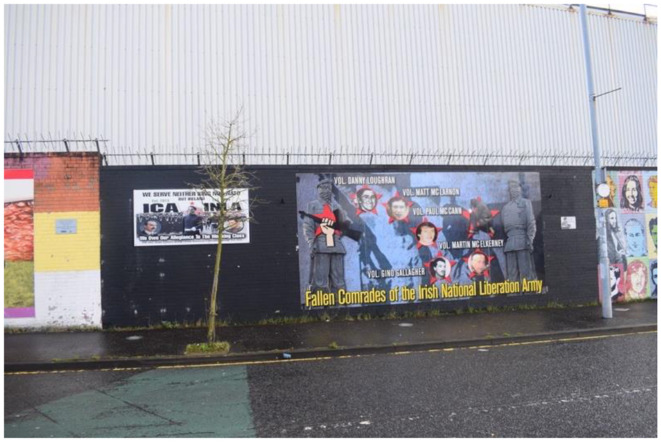
Dissident republican murals of the Easter Rising on International Wall Falls Road, Belfast.

**Figure 3:**
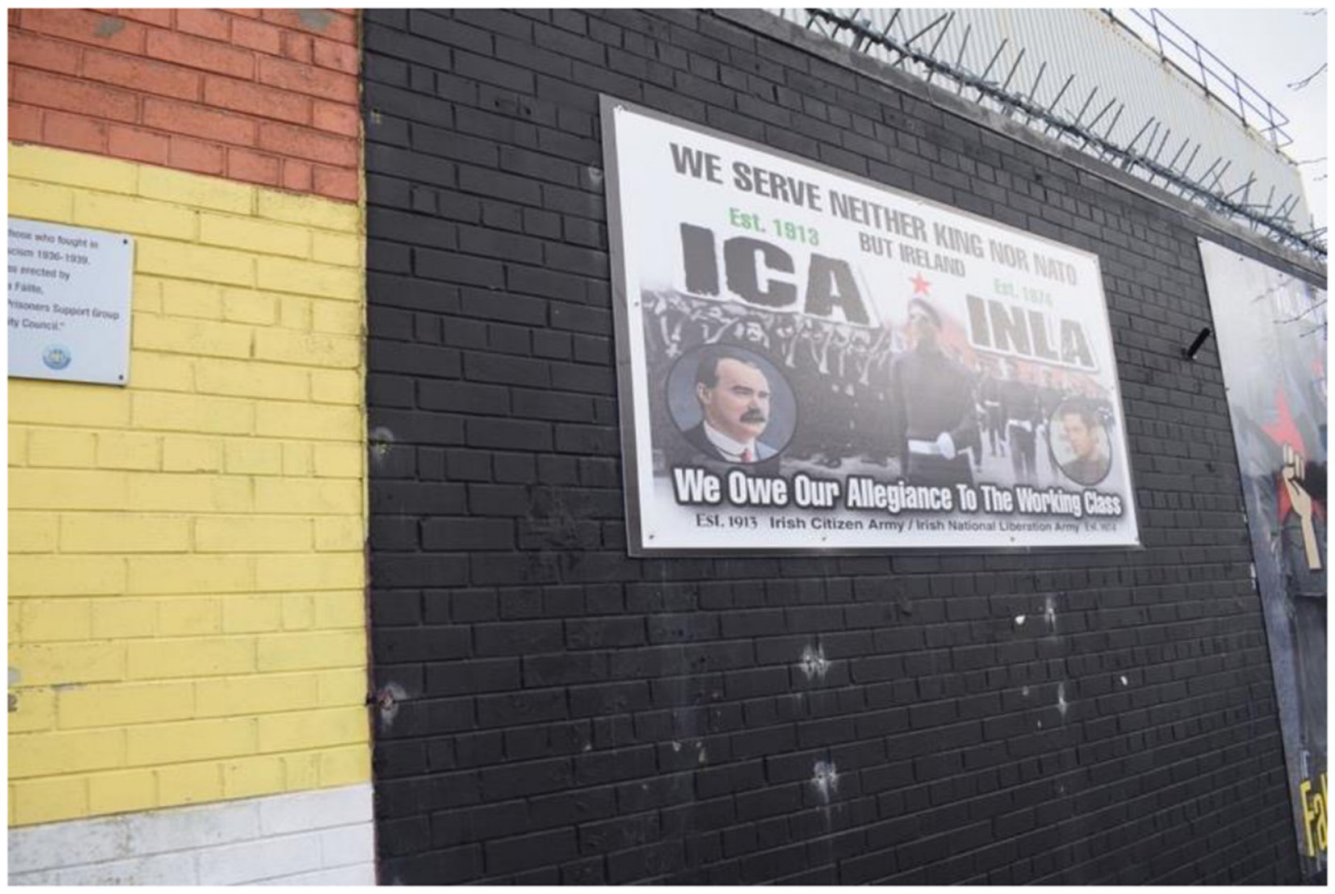
Dissident republican murals of the Easter Rising on International Wall Falls Road, Belfast.

The two principal figures of the Easter Rising, Padraig Pearse and James Connolly, represent distinct dimensions of Irish identity. Pearse embodies the Celtic aspect and is favored by mainstream republicans, while Connolly, who symbolizes the socialist dimension, is favored by dissidents, as reflected in a specific mural. Both dimensions favor the sacrifice as modes to Ireland's unification (Beiner, 2007). Figure 2 features Connolly, his armed group, the Irish Citizen Army (ICA), and the contemporary Irish National Liberation Army (INLA), which claims descent from the ICA. Although INLA was active throughout the Troubles and has since disarmed, some evidence suggests support for the dissident republican movement persists among its members (Viggiani, 2016). Figure 3 depicts the INLA of the Troubles and today. I have chosen to examine both jointly as they engage in a visual dialogue by being situated on the same black section of a wall on the “International Wall”—a site at the intersection of the loyalist Shankill and republican Falls Road. The International Wall traditionally depicts perceived parallels between Northern Ireland's struggle for self-determination and other global conflicts, such as those in Palestine and South Africa. This has changed after Russia's invasion of Ukraine, where the IRSP is supporting Russia, which Figure 2 represents by its headline.

The juxtaposition of these murals highlights the class dimension within republicanism, particularly how working-class bodies bear the brunt of armed struggle. A significant critique from dissident republicans is that mainstream republicans have established a hierarchy within the movement, thus neglecting its socialist ethos (McGlinchey, 2019). The working class is presented as the force uniting Ireland, as evidenced by the central masked Figure in the mural, the headline “We serve neither king nor NATO but Ireland—We owe our allegiance to the working class,” the portrait of Connolly, the ICA members behind him, and the image of Seamus Costello on the right (Figure 2). This interpretation is reinforced by the adjacent mural depicting fallen INLA volunteers, whose portraits appear at various stages of life, suggesting these photographs were taken around the time of their deaths. The mural's background features masked men wielding AK-47s, framed by a red star with the headline “Fallen Comrades of the Irish National Liberation Army” (Figure 3). Moreover, their faces in Figure 2 are positioned within the starry plow, underscoring their working-class credentials. The red star, a symbol of socialism, underscores Connolly's legacy. By emphasizing their working-class identity, dissidents position themselves as the true republican heroes. In the mural to the right, the term “comrades” is employed, emphasizing equality and camaraderie. Within the dissident republican movement, there is no hierarchy among heroes; all are considered equal.

The commemorated individuals are depicted as ordinary men, likely locals, which shifts the narrative away from portraying dissidents as figures of the past or as dangerous men. Instead, they are presented as “the men next door” who fulfilled their duties. This narrative further situates their acts of violence as committed in allegiance to the working class, forging a bond between the dissidents and the community they claim to serve. Moreover, it acknowledges the physical toll of direct violence on their bodies. The dissident interpretation of republican sacrifice diverges from the mainstream narrative, which suggests that the republican spirit protected these men from breaking down. In contrast, a connotative reading of the mural regarding the dark colors, shows how the murals convey a sense of pain, despair, and darker undertones to their message. Thus, illustrating that the spirit transforms republicans into hardened rebels, enabling them to carry out necessary acts of violence. The red color of the stars, stand out meaning that their socialist affiliation made them endure this fight. They are hardening themselves for their community. The spirit, in this context, is depicted as having a malign influence, compelling rebels to undertake actions they would not normally consider. These murals challenge Hariri's concept of “flesh witnessing,” which refers to an understanding gained through bodily experience (Harari, 2009). In the context of republicanism, dissidents have endured the harsh realities of direct violence and do not romanticize it as mainstream republicans do. Their physical and psychological experiences render their bodies sites of collective memory, and it is through their “flesh witnessing” that they assert the correctness of their interpretation.

The combination of these murals creates a linear narrative, tracing how Connolly and the ICA initiated the struggle in 1916, how Seamus Costello and the INLA continued it during the Troubles, and how today's dissident republicans are completing it. This narrative suggests that dissidents of the present and ICA men of the past share the same experiences. It acknowledges the destructive forces of direct violence as a shared experience, but it is not a narrative that offers comfort to those who are broken today; instead, it emphasizes the necessity of enduring these hardships. Unlike the mainstream republican narrative, where the spirit selectively protects some, this narrative suggests that volunteers must brace themselves to become hardened men.

### 3.2 Hunger strike

#### 3.2.1 Mainstream

**Figure 4:**
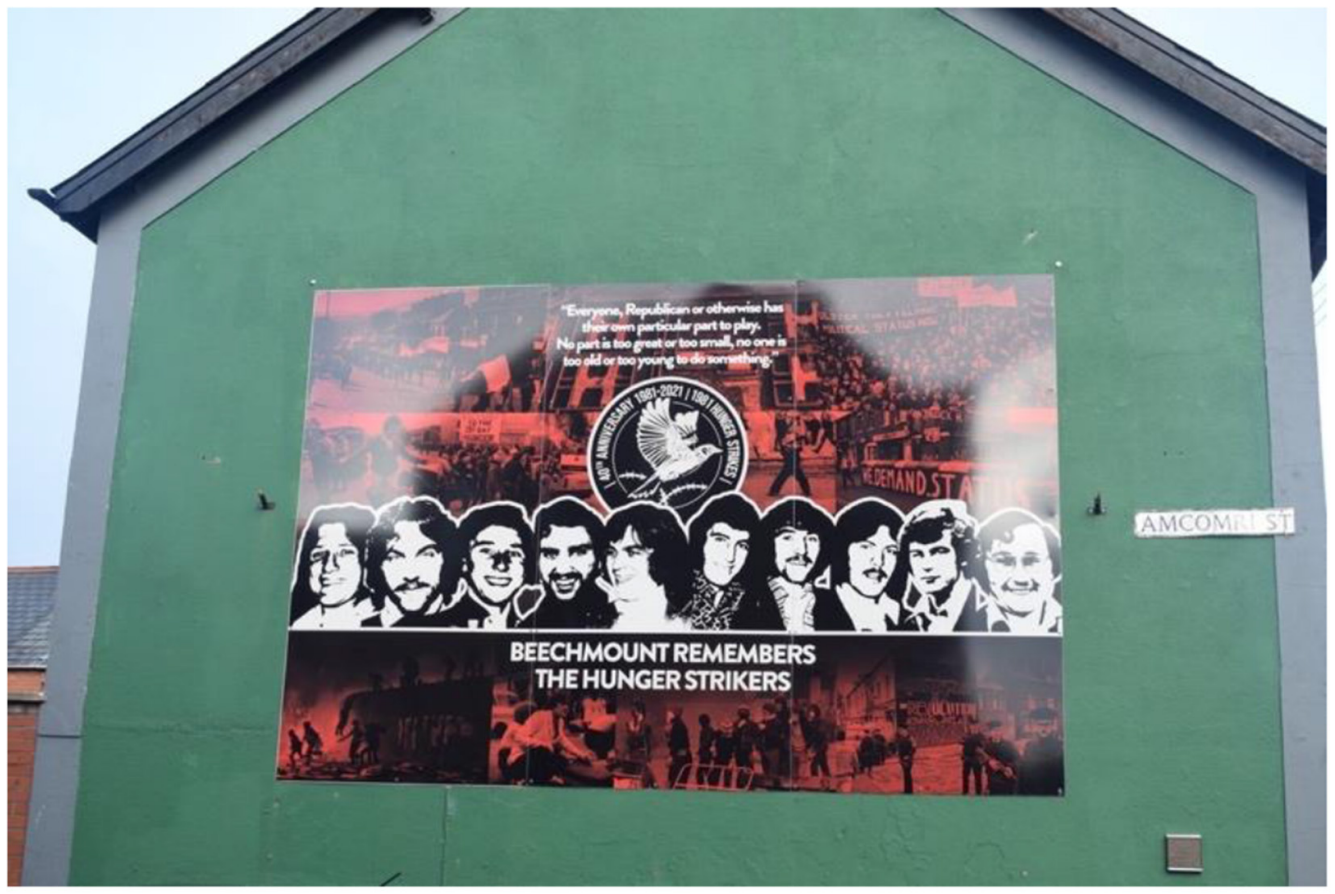
Mainstream republican murals of the Hungerstrike in Beechmount, Belfast.

This mainstream republican mural (Figure 4), located in Beechmount, West Belfast, was erected by residents in 2021 to commemorate the 40th anniversary of the Hunger Strike. The mural conveys a narrative of the unyielding power of the republican movement, a theme reinforced by its background, the depiction of the hunger strikers, and the accompanying quotes. The background features a collage of scenes from the Troubles, including a riot, a gun battle, marching protesters, and police officers, collectively portraying the community's resistance against the British. The Hunger Strike is positioned as the culmination of this resistance, with the hunger strikers prominently placed in the foreground, symbolizing their ultimate sacrifice for the community. Behind the Figure of Bobby Sands, the emblem of the lark—symbolic of both the hunger strikers' sacrifice and the concept of freedom in republican collective memory—reinforces this message. Sands' quote, “Everyone, Republican or otherwise, has their own part to play. No part is too great or too small; no one is too old or too young to do anything,” further underscores the theme of collective effort. The green shade of the house façade, echoing the Irish tricolor, visually reinforces the goal of a united Ireland for which the republicans fought.

The selected dark colors of the mural show a resemblance to the previous analyzed mural: darker undertones to the mural's message. However, the actors in the murals are different: favoring the community over individual volunteers. In this mural's background the community is fighting back and not the PIRA. Thus, the mural has a different donative reading, which impacts the connotative understanding of the mural's message. I will elaborate on these matters in the section on comparison.

The denotative and connotative reading shows how the mural constructs a narrative that links the indomitable strength of the republican community to the bodies of the hunger strikers. Through their sacrifice, these bodies are transformed into symbols of unification. The republicans' continuous willingness to sacrifice themselves is portrayed as the source of their invincibility. This interpretation aligns with Zygmunt Bauman's concept that postmodernity engages with death by utilizing life—life triumphs over death (Bauman, 1992). In this context, sacrifice overcomes death through the metaphor of resurrection. This reading is further emphasized by the mural's red and black tones, which evoke the image of fire, suggesting that the hunger strikers are emerging from the flames. They are portrayed as having risen from the chaos and violence of the Troubles, bequeathing the present to younger generations. The Troubles are thus framed as the rebellion that concluded all rebellions, rendering further republican sacrifice unnecessary and positioning the hunger strikers, and by extension mainstream republicans, as architects of the future.

The decision to create a mural depicting the hunger strikers as sacrificing themselves for the community and future is closely tied to its timing—the 40th anniversary of the Hunger Strike. Collective memory, as Halbwachs argues, comprises selected past events that are interpreted and chosen based on the present (Halbwachs and Coser, 1992). An article in “Republican News” notes that the primary motivation behind the mural's creation was to remember the deeds of the hunger strikers, who are described as “brave.” The organizers emphasize the importance of connecting younger generations to this past, cautioning against drifting too far from it. Rather than criticizing residents for forgetting, they aim to ensure the Hunger Strike is commemorated annually and does not become merely a “mural on the wall” (Republican News, 2021). This perspective underscores how remembrance shapes our connections to the past and how we define ourselves in the present. The past is necessary to structure, construct, and anchor our identities today, though these links are inherently transient, as they are tied to the present (Huyssen, 1994).

The cultural violence embedded in this mural project an expectation that the present must be grateful for the sacrifices made, and if not, it implies a sense of shame. This dynamic introduces a measure of guilt directed at the younger generations. Such questions intersect with broader considerations of how to envision the future and who is included in this vision. Collective memory, grounded in the connections between past and present, must be capable of articulating futures (Huyssen, 1994). The mural's message asserts that Beechmount remembers the hunger strikers and is therefore grateful for their sacrifice, which provided the current reality. However, this raises the question: what is this “now,” and what kind of future will it yield? The societal wounds inflicted by the Troubles and sustained by paramilitary interpretations of the past—upheld through murals such as this—remain unhealed. This ongoing trauma has contributed to a sense of disillusionment among the younger generation, exacerbated by a rampant drug epidemic (Brown and MacGinty, 2003). As mentioned, the makers of this mural did not want it to become a “mural on the wall”, meaning that the hunger strikers' sacrifice would be upheld and continuously remembered. However, this sacrifice is rooted in cultural as it upholds the divide between republicans and loyalists. As it is framed by mainstream republicans as creating the present, one could argue that the hunger strike created the disillusionment. One must ask whether the sacrifice was truly worth it. Should the hunger strikers be remembered if their legacy has led to a present filled with despair? The cultural violence within this collective memory tends to silence such questions, as the surrounding visual culture reinforces the narrative of republican victimhood. It also highlights how cultural violence is not inherent but taught, with these narratives visually transmitted across generations.

#### 3.2.2 Dissident

**Figure 5:**
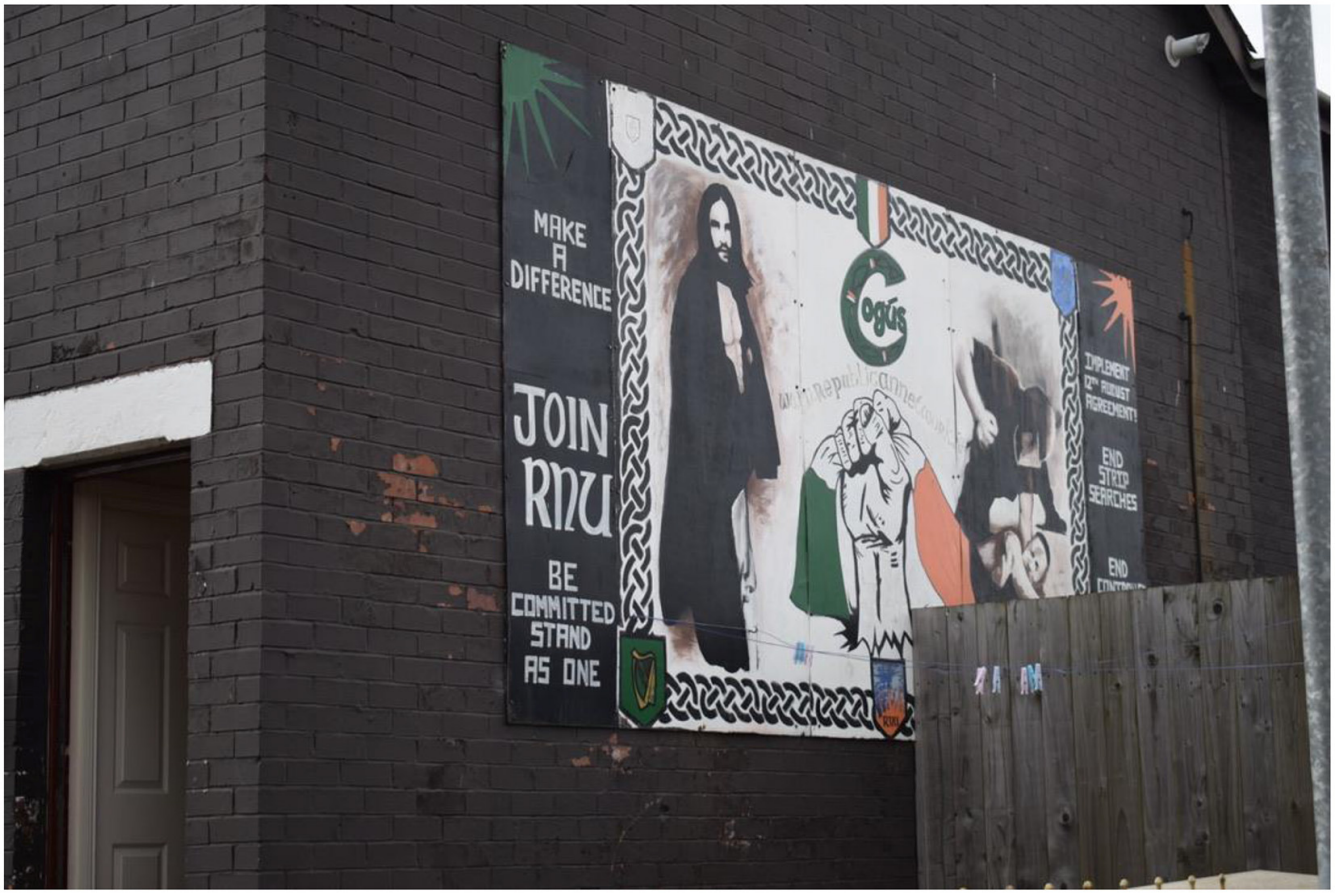
Dissident republican murals of the Hungerstrike in Ardoyne, Belfast.

Ardoyne in North Belfast is a so-called interface area, as it borders the loyalist Shankill and Cliftonville districts (Figure 5). The dissident mural selected for analysis here is notable for its resemblance to the 1980s depictions of the hunger strikers. Commissioned by Cogús, the Prisoner of War (POW) branch of the Republican Network for Unity (RNU), this mural exemplifies the early 1980s Hunger Strike murals. The RNU, a political party formed in opposition to Sinn Féin's support of police reform in 2006, is alleged to serve as the political wing of Óglaigh na hÉireann, a dissident republican paramilitary group. Although the mural is no longer present, its significance lies in the connection it draws between the hunger strikers of 1981 and contemporary dissident republicans.

The mural's symbolism is deeply rooted in the imagery of the 1981 Hunger Strike, featuring motifs such as a man in a blanket, a fist gripping the tricolor, a prison warden beating a prisoner, and a resonant headline. The early murals of the 1981 often depicted the hunger strikers as gaunt men, resembling Christ on the Cross (Rolston, 1998, 2010). These elements collectively suggest that little has changed since 1981. The mural equates the dissidents' sacrifices with those of Bobby Sands in the H-Block and even Christ on the Cross, emphasizing their continued struggle for a united Ireland. This portrayal aligns with Sweeney's (1993) concept of the powerless confronting the powerful, positioning the dissidents as the current underdogs standing against both Britain and mainstream republicans. The dissident prisoners are depicted as bearing the costs of war on behalf of their communities, echoing Olick and Robbins's (1998) assertion that the body serves as a site of collective memory. The mural emphasizes bodily experiences—starvation and physical abuse—as central to this narrative. By highlighting these experiences of direct violence, the dissidents aim to transform the bodies of POWs into symbols of collective memory. However, this does not necessarily mean that the broader republican community perceives them as such, as the interpretation of these symbols can vary. Spillman and Conway caution against overgeneralizing performativity, arguing that it oversimplifies the conditions for its success and overlooks the shared system of collective representations (2007, p. 88). This may explain the mural's deliberate reuse of Hunger Strike symbolism and its focus on bodily experience: it seeks to remind the community of the 1981 hunger strikers' sacrifices and to underscore the continuity of their struggle.

The mural also suggests a vision of the republican community as a unified body, capable of enduring the British occupation through collective strength. The support and endurance of the republican community are portrayed as essential to the sacrifices made by the hunger strikers, framing the community's resilience as a form of sacrifice itself. The mural's call to “Join RNU, be committed, stand as one, and make a difference” reinforces this message of unity and collective action.

The denotative and connotative reading of the mural shows how this mural depicts the community as unharmed by the direct, structural, and cultural violence inflicted by both the British and republican paramilitary groups, which means that the mural scathes over the enduring impact of this violence. The republican societal body was deeply wounded during this period, and this wound remains open. Tomlinson discusses how war and peace bring about significant social changes, noting, for example, the increase in suicide rates in Northern Ireland following the signing of the Agreement in 1998. He concludes that those who grew up during the Troubles and the subsequent “peace babies”^5^ are now the most affected by its long-term repercussions due to the transfer of intergenerational trauma (Tomlinson, 2012). Thus, the sacrifices made by republicans during the Troubles produced a societal wound that is now being sustained and exacerbated by the cultural violence perpetuated through murals. The visual representation of bodily sacrifice in these murals continues to scar younger generations, who struggle to comprehend their pain within the framework of collective memory. Unlike the republican volunteers, these individuals are perceived as weak and, as a result, are viewed as obstacles to the achievement of a united Ireland.

### 3.3 Troubles

#### 3.3.1 Mainstream

In 2009, a memorial was erected in Poleglass, West Belfast, to honor the deceased republican volunteers Patricia Black and Frankie Ryan, as well as politicians Michael Ferguson and Séan Keenan. In 2023, this memorial was further augmented with four murals depicting the deceased. Black and Ryan, who died while on active duty in England due to a premature bomb explosion, are commemorated alongside Ferguson and Keenan, who passed away from natural causes. The murals are situated on a low white wall behind the memorial, with a pathway separating them from the adjacent residential area. The memorial's prominent location along a busy road ensures that its symbolic significance is both visible and accessible to the public. The murals on the right side of the pathway represent members of the Belfast Brigade, while those on the left depict Sinn Féin councilors. This arrangement visually encapsulates the dual pillars of republicanism: the armalite and the ballot box—symbolizing both violence and politics.

The murals themselves are designed as portraits, each accompanied by additional information about the lives of the deceased. The frames of the murals feature the colors of the Irish tricolor and a Celtic pattern. In Black's portrait, she gazes upwards at the viewer with a coy smile, depicted as a loving, kind, and strong-minded daughter, sister, aunt, and friend. She is further commemorated for joining the brigade at the age of 18 out of love for her country, with her determination and dedication earning the respect of her comrades. She is also described as “brave” and a “young woman.” In contrast, Ryan's face is less distinct due to the poor quality of the photograph used. He is described as a man born in England to Irish parents, which gave him a deep understanding of the “injustices committed by the British.” He is portrayed as enthusiastic and brave, having moved to Belfast of his own accord.

The politicians, Ferguson and Keenan, are not characterized by their personal attributes; rather, the focus is on their contributions to the republican cause. Ferguson is commemorated as a soldier, POW, community worker, and MLA. He is described as intelligent, thoughtful, and confident, with tireless energy that served as a dynamo for the area. A nearby roundabout is named in his honor. In his portrait, he is dressed in a suit, exuding a statesman-like presence. Keenan is specifically noted as having been a target for loyalist paramilitary groups, the RUC, and the British Army. He is recognized for his tireless work for Irish language rights, and it is emphasized that he came from a prominent republican family. In Keenan's portrait, the focus is on his face, which is zoomed in, with him looking away from the camera.

The design of the memorial site—including the memorial stone, memorial wall, and murals—reveals an underlying tension within republicanism regarding the notion of political engagement as a form of sacrifice. Traditionally, sacrifice has been closely associated with direct experience and exposure to violence. The murals are positioned behind the memorial wall and stone, with the wall designed as a plaque that places the names of Ferguson and Keenan at the center, flanked by armed republican volunteers and the four shields of Ireland. The wall is colored in black, gold, and green. The memorial stone, however, only mentions Black and Ryan, who are specifically identified as “Óglach,” meaning volunteer or soldier. The stone's placement in front of the wall creates the illusion that the volunteers are flanking Black and Ryan, saluting them in death. This arrangement also partially obstructs the view of Ferguson and Keenan's names, subtly suggesting that violent sacrifice is considered more significant than political service in the republican collective memory.

The connotative and denotative reading of the mural's emphasis on violent sacrifice shows the implications for how to understand Northern Ireland today. In mainstream republicanism, the belief is that politics will eventually lead to the unification of Ireland, implying that there is room for political sacrifice. The biographies of Ferguson and Keenan also highlight their extraordinary contributions to the republican cause, portraying them as figures filled with energy and the republican spirit, enabling them to achieve more than the average individual. This narrative offers an alternative interpretation of what constitutes republican sacrifice and its effects—depicting it as a form of energy that drives individuals to extraordinary accomplishments.

However, the design of the memorial site ultimately suggests that the sacrifices of Black and Ryan are valued more highly than the political contributions of Ferguson and Keenan. This aligns with Jarman's concept of murals that lend, withhold, and sometimes silence meanings. The visual culture produced by the murals communicates a hierarchy within the republican collective memory, where involvement in direct violence is prioritized over political engagement. The cultural violence has shaped collective memory to emphasize the use of direct violence to achieve the unification of Ireland. In the context of contemporary Northern Ireland, this perspective elevates those involved in the Troubles, as they are seen as having facilitated Ireland's unification. At the same time, it lends credence and legitimacy to the dissident interpretation that violence remains necessary.

The republican collective memory's focus on death and violence as tools for unifying Ireland both supports and challenges Heath-Kelly (2016) argument that security and war efface death while paradoxically using it to create the illusion of a nation. Heath-Kelley contends that sacrifices are deeply intertwined with the identities of political communities and their survival, but that death itself is masked through language, discourse, and performance. In this case, violent death is portrayed as the foundation for a future united Irish nation. While the memorialized individuals are physically dead, their sacrifices continue to live on. Ryan and Black, through the premature bomb, are remembered not as victims of a mistake but as participants in the armed struggle, their deaths transformed into sacrifices. The politicians, though not killed in active service, are portrayed as having lived lives so deeply embedded in republicanism that their natural deaths are also framed as sacrifices, the culmination of a lifetime of dedication to the cause.

#### 3.3.2 Dissident

West Belfast, particularly Falls Road, is considered the heartland of republicanism. Historically, dissident republican murals were marginalized within republican spaces in Belfast. However, by 2018, dissident groups began asserting their presence by situating their murals near, or alongside, mainstream republican murals. This spatial juxtaposition has fostered a form of visual warfare between factions, contesting the interpretation of republican sacrifice (Larsson, 2021). Upon my return to Belfast in 2023, I observed that this process had intensified, exemplified by two specific murals located near a Garden of Remembrance dedicated to those who served in the PIRA. Both murals bear the signature of the Republican Network for Unity, a dissident republican organization. I have chosen to analyze these murals together due to their shared focus on the Falls Road Curfew, which took place from July 3rd to 5th, 1970. The Curfew began as a British Army operation to search for weapons but escalated into a gun battle between British forces and the PIRA. To quell the violence, the British Army imposed a 36-hour curfew, which was ultimately broken by women from neighboring republican areas who entered Falls Road with food and supplies. The British Army's handling of the event galvanized many Irish Catholics to support the PIRA (English, 2003; Taylor, 1997). These murals were likely created to commemorate the 50th anniversary of the curfew's end in 2020.

**Figure 6:**
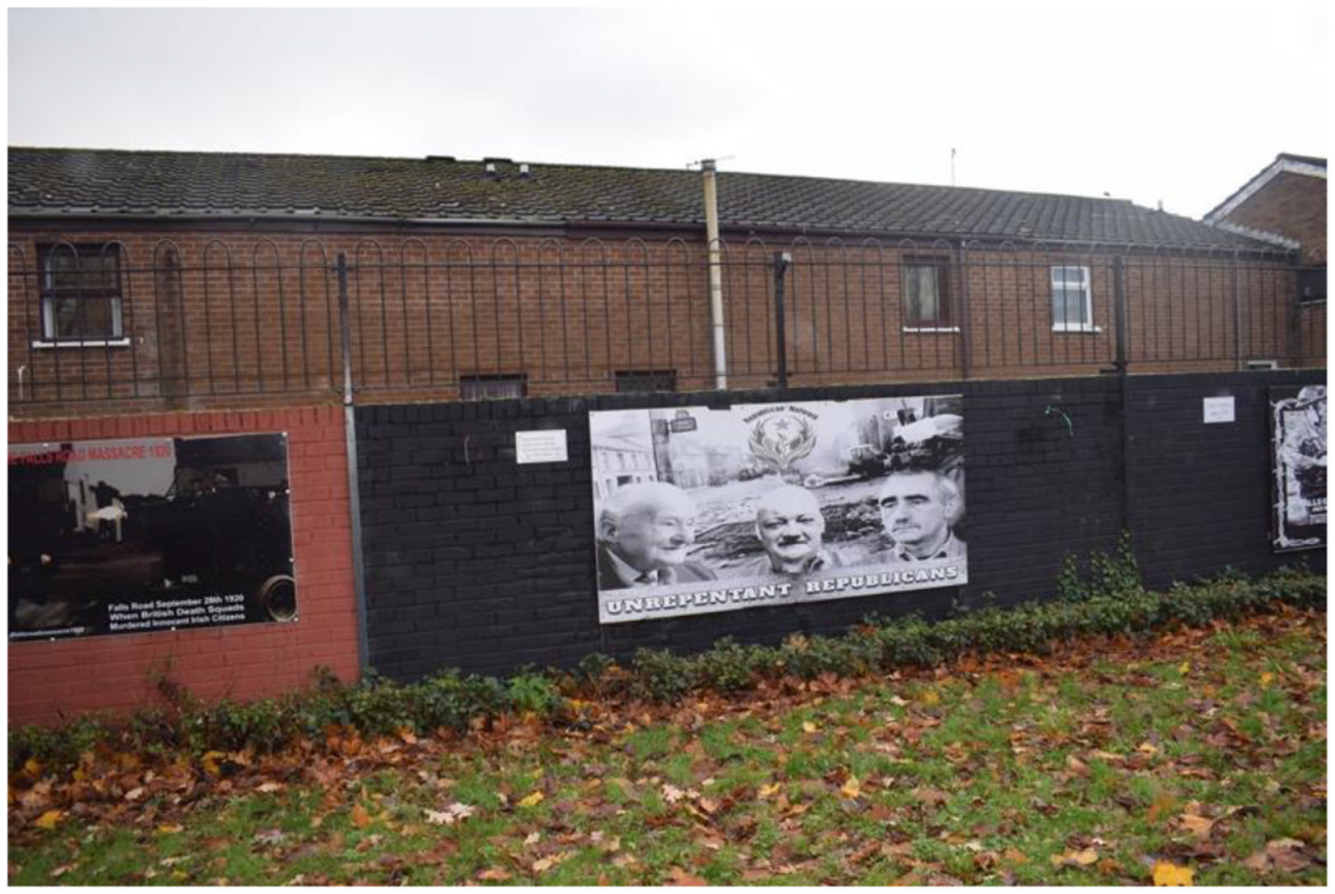
Dissident republican murals of the Troubles on Falls Road, Belfast.

**Figure 7:**
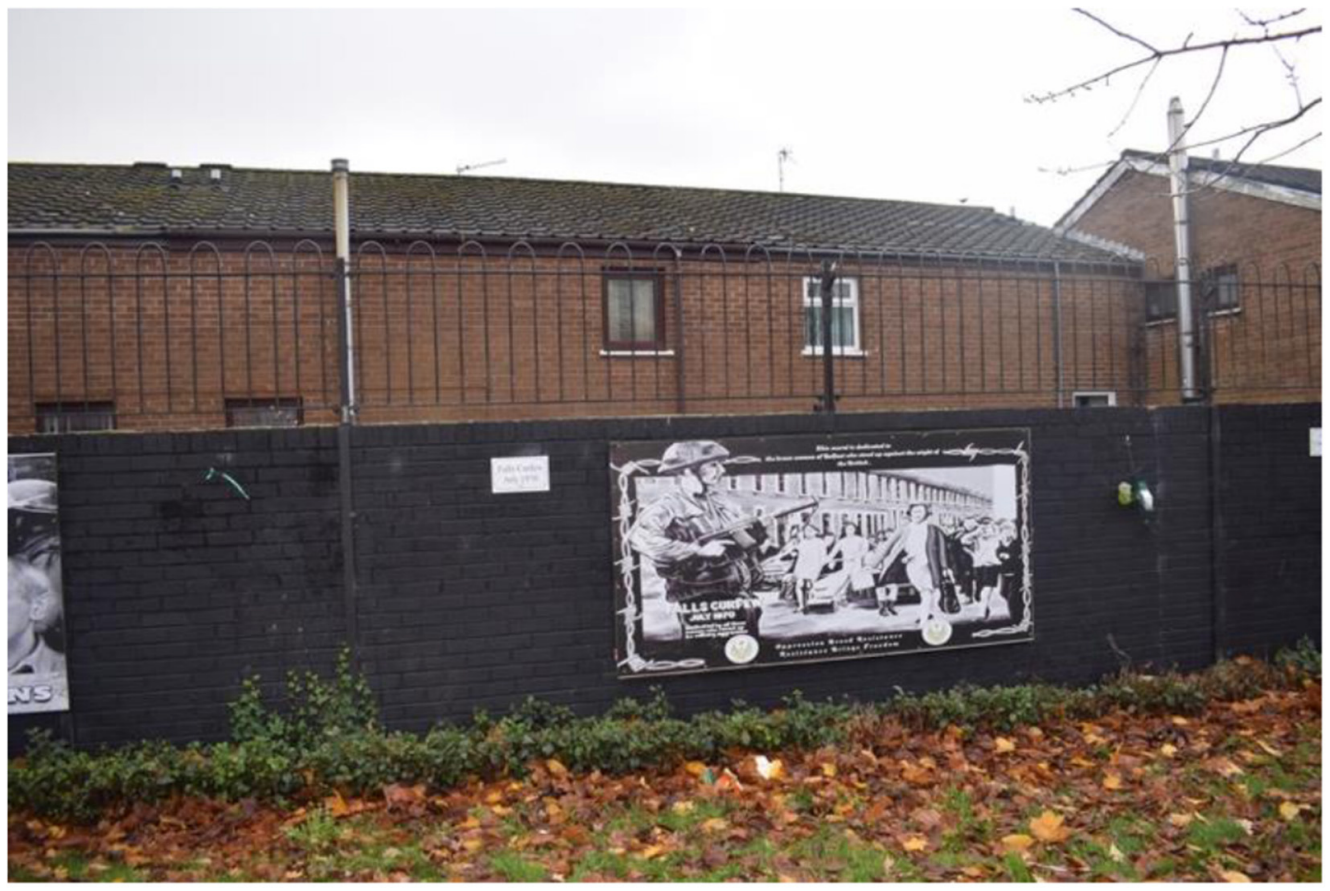
Dissident republican murals of the Troubles on Falls Road, Belfast.

In the previous denotative and connotative readings of mainstream murals, republicanism was found to be expressed as a spirit manifesting through individual action. As seen here on the mural (Figure 6) depicting Billy McKee, Brendan Hughes, and Alec Murphy and the mural dedicated to the women of West Belfast who ended the curfew (Figure 7). These murals continue this theme of individual action by portraying the republican community as comprising both men and women of action. However, the agency of the community must be juxtaposed with the depiction of three “unrepentant republicans” in Figure 6. All three were prominent dissident republicans and members of the Belfast Brigade who participated in the gun battle during the curfew. Behind these men is a snapshot of Falls Road in 1970. The mural of the women (Figure 7), depict the women's agency differently than the men. The visual presentation of these murals differs; the mural depicting men is composed of photographs, while the women's mural is a drawing. Moreover, in the foreground of the mural of the women, a British soldier is depicted as the women march towards him. The anchoring message of the women's mural reads: “Dedicated to the brave women of Belfast who stood up to the might of the British—dedicated to all those who faced up to military aggression—oppression breeds resistance—resistance brings freedom.” Unlike the men, the women are drawn rather than photographed, making it impossible to identify any individuals (Ashe, 2012; Ashe and Harland, 2014). The denotative and connotative reading of the murals uncover how this mural emphasizes the resilience of the republican community, with the women portrayed as united and determined in their resistance against the British soldier. However, the contrast between the use of photographs for the men and a drawing for the women suggests a hierarchy of value, where direct violence is prioritized over non-violent protest. While the murals acknowledge that the women's actions supported the volunteers' bodily sacrifices, the women are not recognized as individuals. Instead, they are almost mythologized, depicted as extraordinary figures coming to the rescue of the men. This portrayal engages in a form of cultural violence, erasing the agency of the women by presenting them as mythical beings rather than ordinary individuals. Thus, failing to represent their individual sacrifice.

As reflectors of collective memory and identity, murals correspond to contemporary contexts. The narratives within these murals resonate with the curfews imposed during the COVID-19 pandemic. During the pandemic, there were protests within the republican community against restrictions, particularly those concerning funerals. Although these restrictions affected everyone in Britain, the republican community interpreted them as an attempt by the British government to stifle republican freedom of expression (O'Neill, 2020). Prior to 1998, funerals were one of the few public occasions where individuals could openly express their Irish and republican identity. Funerals were moments when a person's membership in the PIRA was publicly acknowledged. By creating these murals, a visual connection is drawn between the British Army's actions during the Troubles and the contemporary measures enforced by the PSNI during COVID-19. This visual culture suggests that the COVID-19 restrictions were a continuation of British efforts to suppress the republican community and their right to express their Irish identity. Although the COVID-19 restrictions were not as coercive as the British Army's measures during the Troubles, the murals' narratives equate them. This scenario opens a discussion about how collective memory is negotiated within a group. In this case, while mainstream republicans and Irish Catholics may not perceive the COVID-19 restrictions as equivalent to past coercive measures, dissidents are attempting to portray them as such. This narrative implies that republicans must continue to resist British authority, framing the present as an extraordinary time that demands protest (McGlinchey, 2021; Woodhouse, 2020).

This encouragement of action allows dissidents to position themselves as the true republicans. As mainstream republicans engage in power-sharing, they are portrayed as complicit in suppressing the republican cause. In contrast, dissidents are depicted as resisting both British authority and mainstream republicanism, striving to elevate the republican movement. This power dynamic is underscored by the background of the first mural, which features a contemporary snapshot from the 1970s, portraying Belfast as a war zone. This depiction serves to legitimize the men who fought at the time as soldiers rather than terrorists, reinforcing the dissident republican visual culture that highlights the realities of direct violence to validate the actions of republicans during the Troubles.

### 3.4 Comparison

This article examines the representation and interpretation of wounds within the republican collective memory and identity, focusing on how these wounds either facilitate or impede the realization of a united Ireland. Through an analysis of republican murals, this study investigates the portrayal of the costs of war across three pivotal events: the Easter Rising, the Hunger Strike, and the Troubles. In this section, I will first discuss the differing depictions of the volunteers, subsequently discuss how the bodily sacrifice of the volunteers is framed, followed by an exploration of the societal bodily sacrifice. I will then examine the interconnection between these two forms of sacrifice. Finally, I will address the preference within the republican collective memory for bodily suffering over mental struggles.

In the denotative and connotative readings of the mainstream and dissident visual cultures, there is a similarity regarding the position of living and deceased volunteers. Deceased volunteers are remembered with their names and living volunteers are depicted as masked men. This is especially evident in the dissident visual culture. This is a continuation of the republican tradition of not revealing your membership in the PIRA until after death. Funerals are traditionally the moment where the membership was revealed to the world (Viggiani, 2016). In the mainstream visual culture, all faces and identities are visible to the viewer; meaning that no new volunteers are needed. They have become canonized within the republican collective memory through their sacrifices.

**Figure 8:**
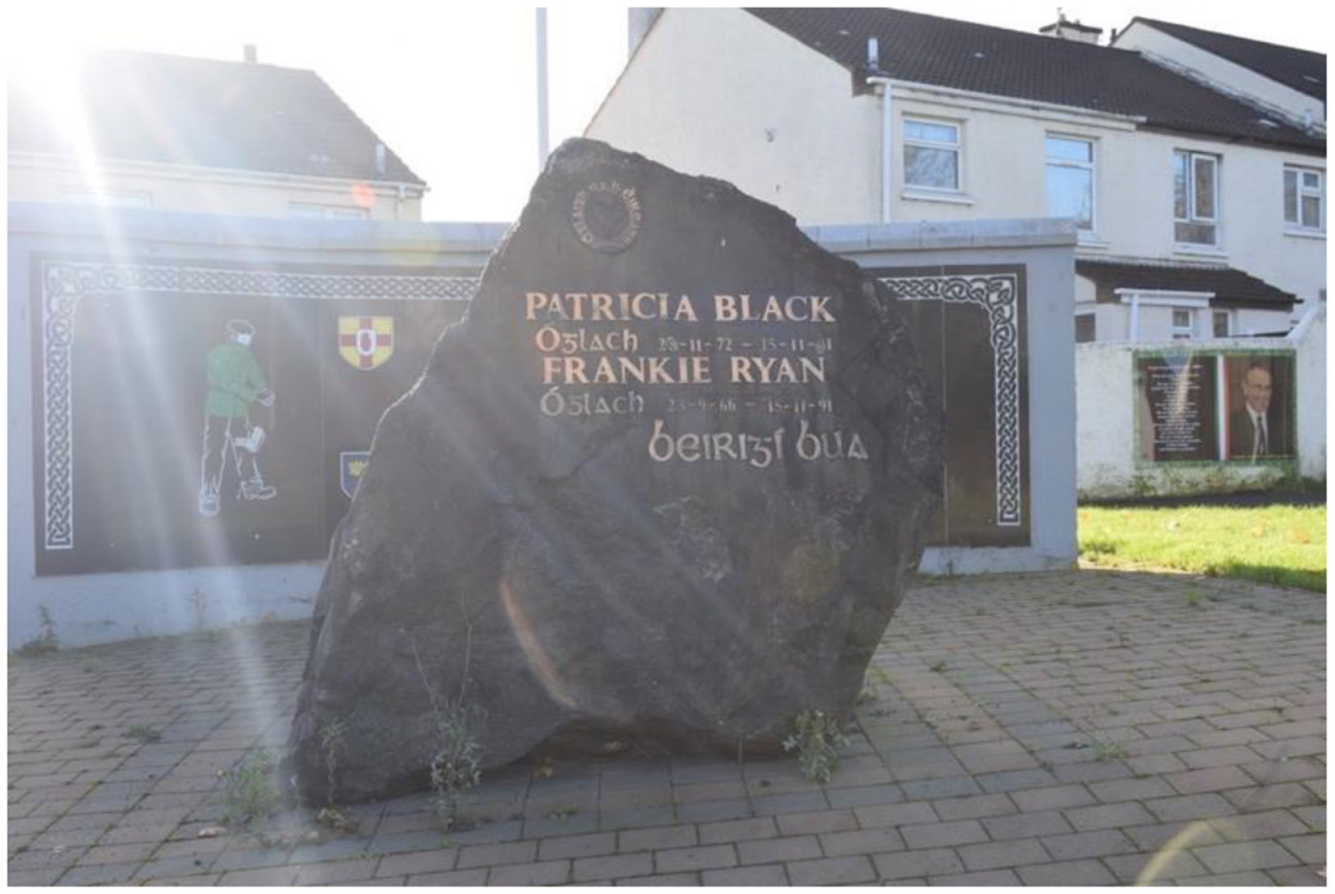
Mainstream republican memorial of the Troubles in Poleglass, Belfast.

**Figure 9:**
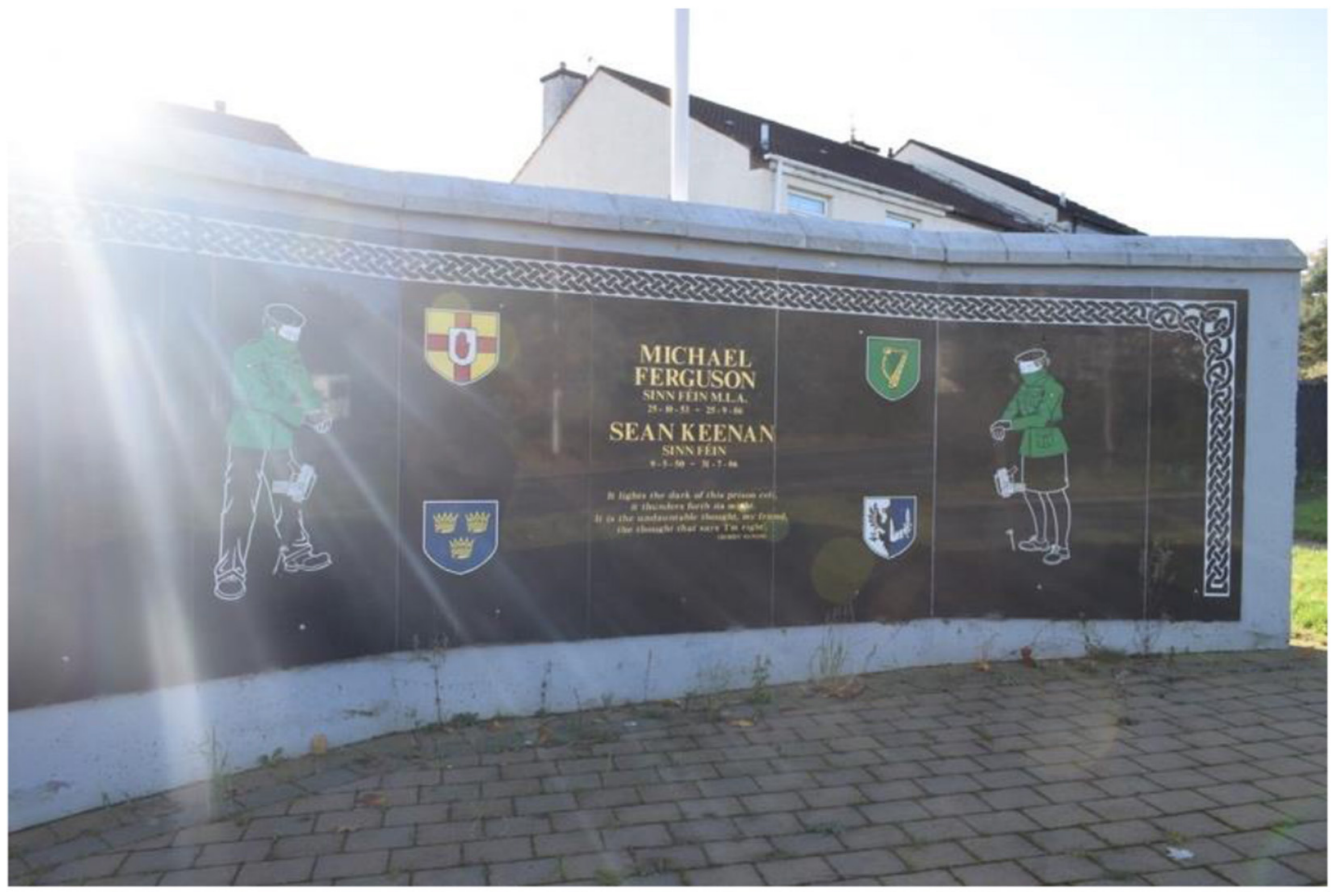
Mainstream republican memorial of the Troubles in Poleglass, Belfast.

**Figure 10:**
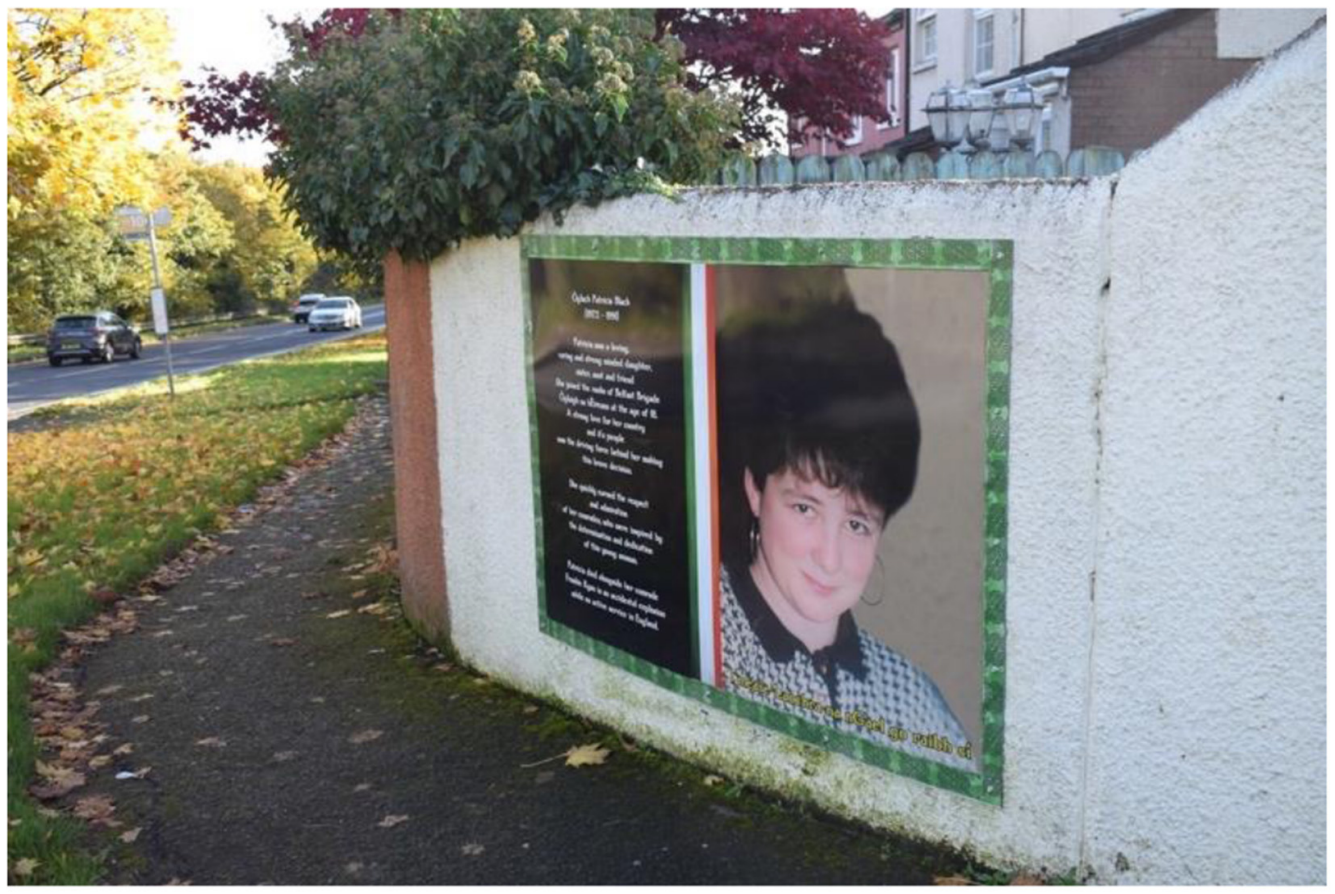
Mainstream republican memorial of the Troubles in Poleglass, Belfast.

**Figure 11:**
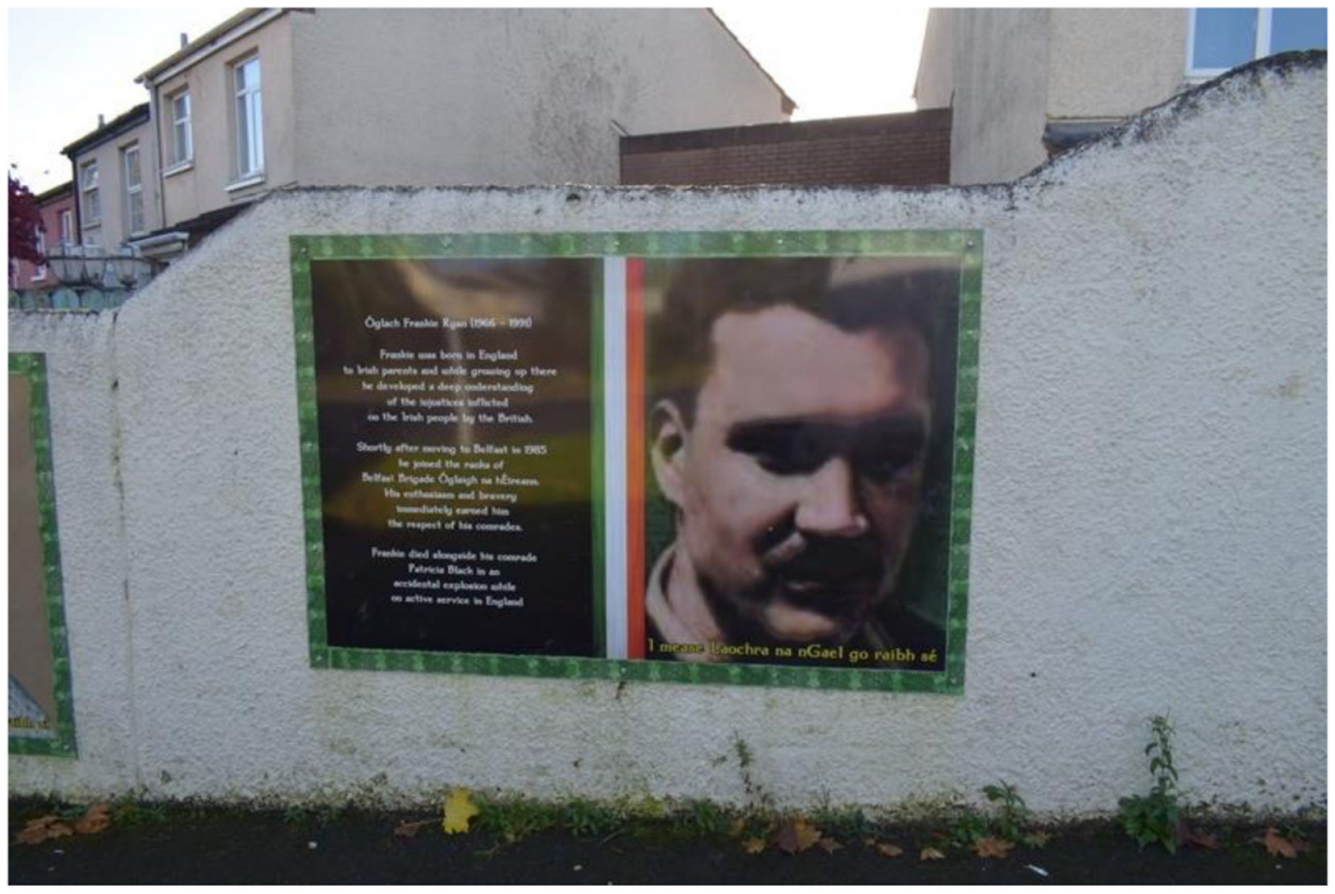
Mainstream republican memorial of the Troubles in Poleglass, Belfast.

**Figure 12:**
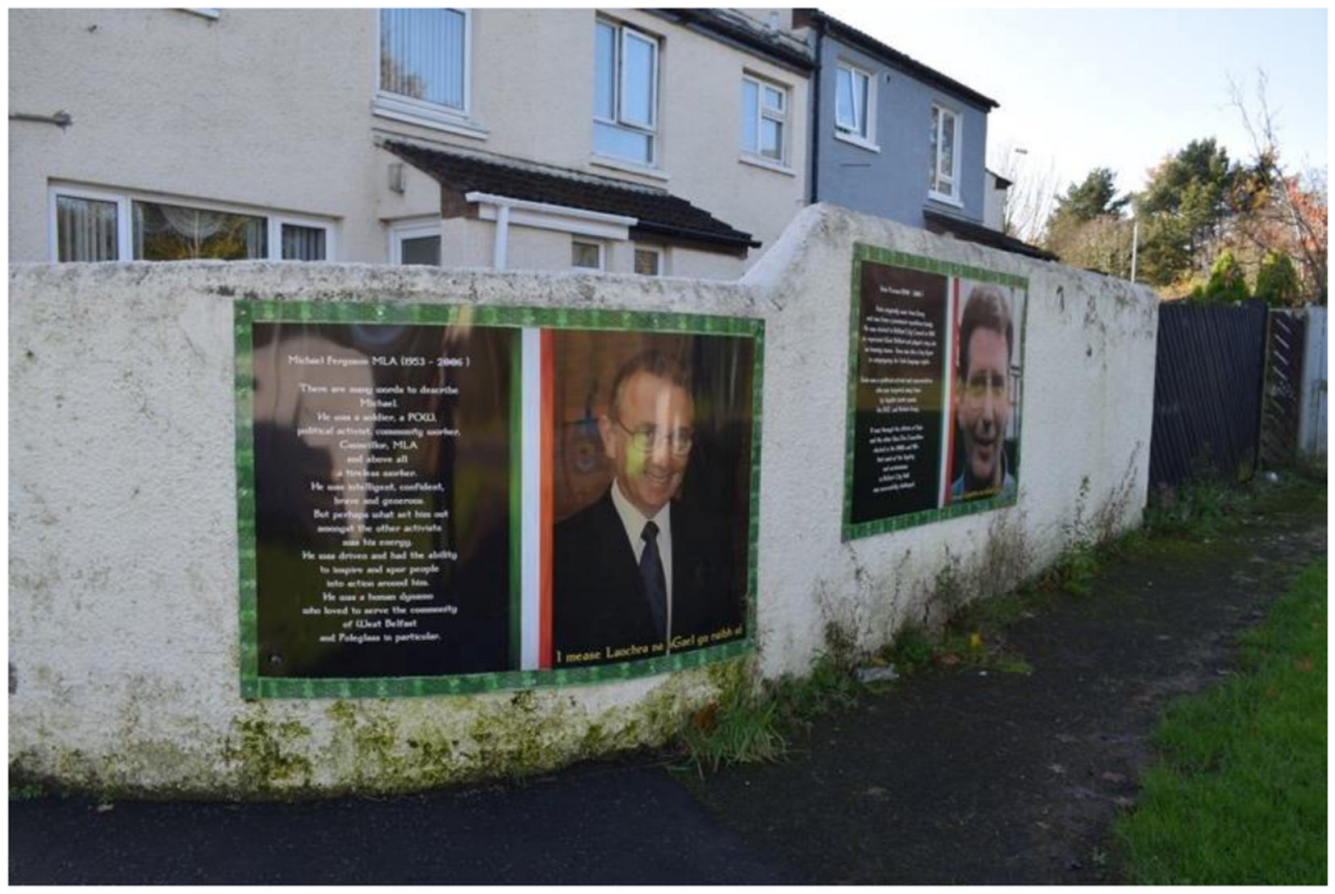
Mainstream republican memorial of the Troubles in Poleglass, Belfast.

**Figure 13:**
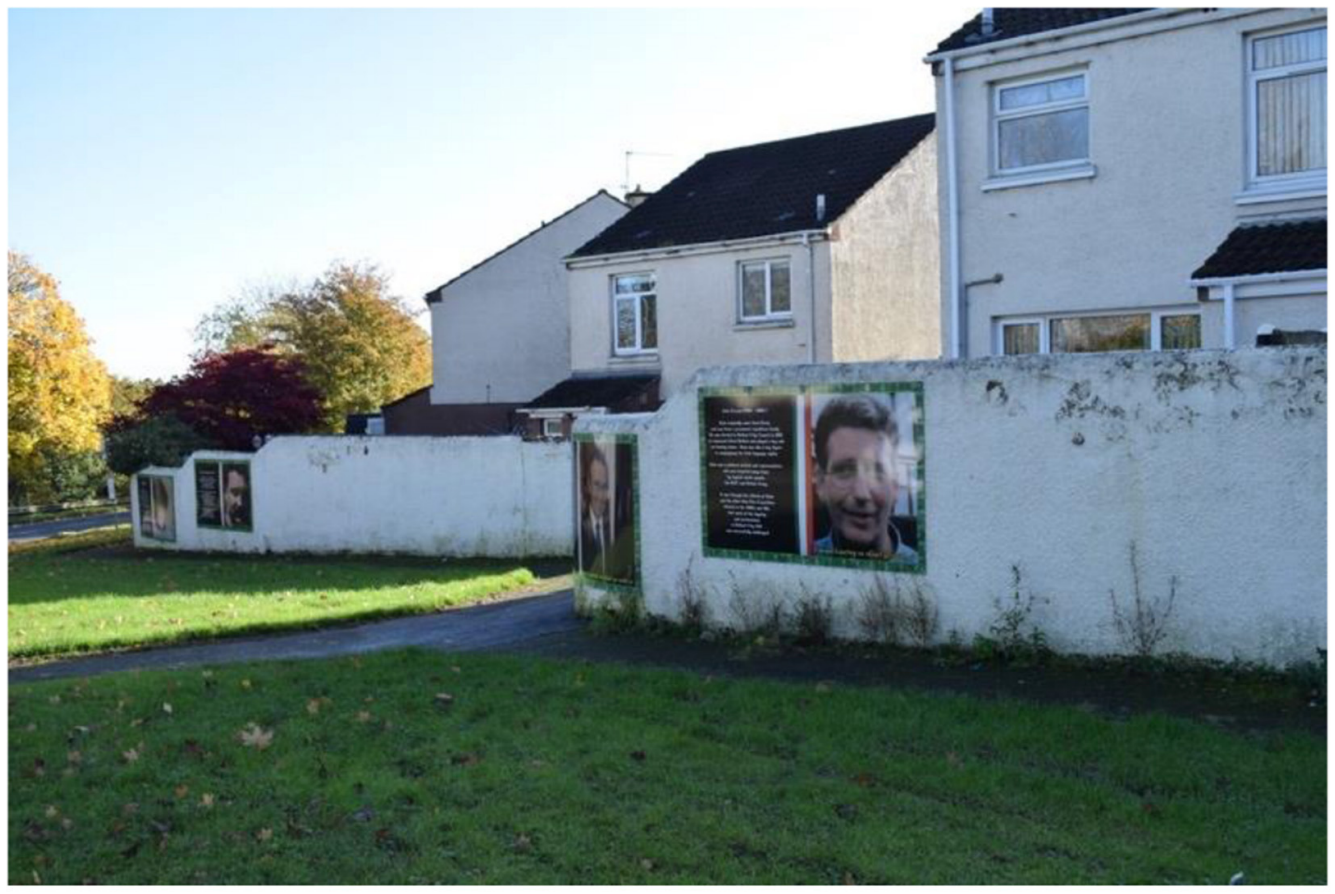
Mainstream republican memorial of the Troubles in Poleglass, Belfast.

This study has examined how bodily sacrifice is portrayed in republican murals. This involved primarily analyzing how paramilitary groups have highlighted their own involvement in the conflict, as they perceive their actions as sacrifices made for Ireland. The denotative and connotative reading of the rebel, the individual who sacrifices themselves, is that of a fighter. It is embedded in the narrative that they are martyrs for Ireland, as this is part of the Irish collective memory. However, the presentation of martyrdom and the symbols used to convey it vary significantly. The connative and denotative readings of the murals reveal that both dissident and mainstream republican visual cultures frame sacrifice primarily through bodily experiences, such as enduring or inflicting direct violence, or participating in forms of protest, such as marches. In both contexts, violent sacrifice is privileged over non-violent forms of sacrifice. However, the analysis identifies two distinct forms of bodily sacrifice within this visual culture: the individual sacrifice of volunteers and the collective sacrifice of the community. These sacrifices are interlinked, with the community's sacrifice reinforcing that of the volunteers. Nonetheless, significant disparities exist in how these sacrifices are understood and depicted, leading to divergent portrayals of the costs of war. In dissident portrayals, the potential to become a rebel remains viable, whereas it is absent in mainstream republican visual culture. Although political figures are mentioned in the Poleglass mural (Figures 8–13), their sacrifices are not depicted as being as significant or honorable as the violent sacrifices. These contrasting portrayals consequently impact the republican community and Northern Irish society in differing ways.

The light and vibrant colors used in mainstream murals depict a romanticized version of sacrifice, in contrast to the dark hues of dissident murals, which emphasize the harsh realities of sacrifice. These contrasting visual representations reflect differing understandings of the rebel identity and the transformation of volunteers; how sacrifice affects the volunteer's body and the role of the republican spirit in this transformation. In mainstream republican visual culture, the republican spirit is depicted as shielding the volunteer from the physical toll of sacrifice, suggesting that the volunteer emerges from the experience stronger and unharmed, akin to a phoenix. In this narrative, the spirit's protection implies that if a volunteer is affected by physical or psychological ailments, they were not truly a rebel, as they lacked the spirit's protection.

Remembrance is intricately linked to contemporary needs, with visual representations crafted to explain and justify the present rather than to accurately depict historical events. While both mainstream and dissident republican visual cultures portray these rebellions to project their respective viewpoints, dissident representations do not sanitize the direct violence of these rebellions as the mainstream does. For example, the mainstream Hunger Strike mural in Beechmount (Figure 4), through its use of black and red, conveys the unsettling nature of living through the Troubles, yet simultaneously obscures the specific events of the Troubles, rendering the past ambiguous. This ambiguity complicates the understanding of the context from which the hunger strikers emerged. Close examination of these murals reveals the everyday experiences of violence—marches, violent protests, and the community's support for the hunger strikers—while omitting depictions of PIRA actions, such as masked gunmen. This omission allows mainstream republicans to sanitize the realities of the Troubles and the armed struggle. As cultural violence through language may sanitize direct violence, legitimizing “our” violence over “theirs” (Galtung, 1990). These visual snapshots align with the mainstream republican narrative, which integrates the IRA into the community, portraying civilian resistance as part of the armed struggle. By not depicting their own direct violence, mainstream republicans position themselves as blameless victims of British oppression (Mitchell, 2015). Prior to 1994, republicans portrayed the IRA's violence as distinct from the community, emphasizing their own capital in direct violence (Larsson, 2021). The cultural violence underlying this presentation positions the PIRA as protectors of innocent victims, legitimizing their actions within the Stormont.

This reasoning establishes a hierarchy within mainstream republican collective memory and identity, where those involved in the armed struggle and capable of concealing their suffering are elevated to an elite status: the heroes who shaped the present. Moreover, as the armed struggle has ceased in mainstream republican interpretation, there is no longer an avenue for new generations to join this elite. However, within the mainstream republican identity, it remains possible to achieve sacrifice through political engagement. In this interpretation, bodily sacrifice is redefined as tireless dedication to the republican cause, with the spirit protecting the volunteer from fatigue. Consequently, those not working toward Irish reunification are projected as lazy and lethargic.

The denotative and connotative readings of the dissident murals reveal how the dissident visual culture offers an alternative hierarchy, wherein the armed struggle continues, allowing dissidents to still aspire to become rebels. The murals of the Easter Rising and the Troubles introduce a class dimension to dissident visual culture, portraying rebels as working-class men, in contrast to mainstream republicans, who are depicted as abandoning their working-class roots by working in the Stormont. Dissident visual culture emphasizes the realities of direct violence, highlighting the actions required during these rebellions and what it means to be a rebel. The hardening of the dissidents' bodies is portrayed as integral to their working-class identity. This is conveyed through the dissidents' use of dark colors, focus on weaponry, and depiction of dissidents as men of direct violence in their murals. The dissidents' sacrifice is twofold: first, by fighting against the British and willingly subjecting themselves to violence, and second, by leaving civilian life behind. The dissident rebel is thus portrayed as someone who no longer lives within the community but willingly resides in the shadows.

This interpretation and portrayal could encompass individuals suffering from PTSD; however, such reasoning is detrimental as it may prevent those affected from seeking necessary assistance. When the analysis shifts to those within this visual culture who are impacted by the aftermath of the Troubles, there is an implicit acknowledgment of the destructive effects of direct violence on both body and mind. Yet, dissident republicans are not depicted as experiencing these repercussions; they are portrayed as being beyond the capacity to feel such destruction, implying that those who do feel the repercussions of the Troubles are excessively human.

Both factions, in their visual cultures, emphasize that the violent bodily sacrifice of the volunteer is dependent upon community support. This depiction is historically significant, as neither the Easter Rising, the Hunger Strike, nor the Troubles initially garnered widespread support; such support emerged only after the sacrifices were made. Nevertheless, in these visual cultures, it is portrayed that for volunteers to endure the impact of their sacrifices, they must be supported by the community. Mainstream republicans depict a supportive community that facilitated the volunteers' transformation, while dissidents portray the necessity of ongoing support as they continue to sacrifice themselves for the community. The support, therefore, is depicted as either easing or exacerbating the transition from volunteer to rebel. In mainstream depictions, the community is shown as providing an atmosphere that nurtures the volunteers, allowing them to flourish into rebels. In contrast, dissidents illustrate a community withholding support, thereby intensifying the pain of sacrifice.

In medical sociology, the body is viewed as an agentic subject, capable of transforming society. The individual bodily sacrifices of volunteers are portrayed as facilitating the eventual unification of Ireland. Within the broader republican visual culture, sacrifice may also be a communal experience: the community acts as a single body, engaging in acts of resistance against the British and offering support and relief to those who are fighting. This theme is evident in both dissident murals of the Troubles and mainstream depictions of the Hunger Strikers. The differences between these depictions are minimal, as both emphasize the agency and actions of the community. Sacrifices are depicted through non-violent protests, such as marches, where individuals or groups willingly expose themselves to potential harm. This broadens the concept of sacrifice to include actions where individuals or groups place themselves in danger or engage in non-violent forms of resistance.

However, it is the individual violent sacrifice that takes precedence over the communal. This is evident in how individual sacrifices are highlighted in the murals, or how specific individuals are chosen to represent an entire rebellion. These figures are often surrounded by symbolism that situates them within the framework of Irish identity, thereby transforming their deaths into sacrifices for Irish unification. This focus on individuality aligns with Narváez's (2012) argument that collective memory studies should consider the body as a site where the past becomes embedded, both individually and collectively. Spillman and Conway (2007) contend that this approach can oversimplify the conditions for the success of performative acts and neglect the shared system of collective representations. This article demonstrates that murals play a crucial role in transforming actions into sacrifices. The bodies of individual volunteers become canvases of the past, with their experiences performing the past. By imbuing their deaths, experiences of direct violence, and incarceration with symbols of rebellion and Irish identity, these acts are reinterpreted as sacrifices for Ireland. The volunteers enact the past through their bodies; however, they cannot be recognized as rebels without the attachment of symbolic meaning and the external validation of their sacrifice as legitimate. This conclusion can only be reached by understanding the republican volunteers' bodies as cultural canvases, where physical experiences are visually expressed and clothed in cultural meanings.

In this article, collective memory is conceptualized as a negotiation among members of an identity group. The determination of who is recognized as a rebel and as sacrificing themselves for the community results from this negotiation. As demonstrated, not all experiences are categorized as sacrifices, thereby supporting Narváez's (2012) observation that meanings may be withheld. While dissidents frame their actions as sacrifices, they receive little support in contemporary Northern Ireland (McGlinchey, 2019). However, the idea that meanings and support can be withheld also applies to those suffering from trauma related to the Troubles (Bunting et al., 2013). Their suffering could be interpreted as inflicted by the British, thereby transforming their pain into an ongoing sacrifice for Irish unification. Nevertheless, this interpretation has not emerged due to prevailing narratives of the resurrected rebel and the hardened rebel.

By juxtaposing these two visual cultures, it becomes apparent that in post-conflict republican Northern Ireland, it is not the sacrifice itself that holds significance, but rather how the volunteer *mentally copes* with the aftermath of the sacrifice. The emphasis is on the volunteer's ability to suppress and not display the psychological and physical repercussions of their sacrifice. This rejection of those suffering mentally from the Troubles underscores how the republican collective memory prioritizes bodily actions over mental struggles. The Hunger Strikers, for instance, engaged in mental resistance by speaking only Gaelic to their wardens, yet speaking is still a form of action (Fierke, 2012). Mental suffering, which often manifests through stillness, fatigue, and numbness, does not align with the republican spirit that valorizes men of action: rebels. This conclusion arises from the study of how republican sacrifices have been portrayed across three rebellions. In the denotative and connotative readings of the murals, I have found that republican bodies serve as canvases for the collective memory, where their actions are embedded and understood as extensions of republican history. In terms of performativity, the depicted volunteers not only act out the republican memory but embody it. If one is incapacitated by mental health issues, one cannot perform the collective memory.

This emphasis on action extends to the societal body. As noted, the actions of the community are crucial in protecting and supporting the volunteers. If the community is unable to act, the volunteers cannot effectively carry out their violent resistance. Therefore, it is essential that the community remains capable of action. A community overwhelmed by individuals unable to act cannot facilitate the unification of Ireland. This need for action is reflected in how republican paramilitary groups punish those involved in drug use (Morrison, 2020). One consequence of the trauma from the Troubles is the previously mentioned heroin epidemic, which has significantly impacted both sides of the peace walls in Northern Ireland (Long, 2021; Boland et al., 2020a,b). Some republican and loyalist paramilitary groups contribute to the problem by selling drugs, while others fight against drug dealers (Viggiani, 2016). Despite their differences, these groups commonly use direct violence to control drug users and sellers, maintaining their authority through the threat of violence. In this way, punishing drug users and dealers is seen as a means of preserving the societal body and ensuring it remains ready to act for the republican cause.

## 4 Conclusion

This article has examined how the cost of war is portrayed in mainstream and republican murals, through denotatively and connotatively analyzing the representation of bodily sacrifice in three rebellions. Mainstream republicans depict the cost of war as a minor price, justified by the preservation of the republican spirit. This portrayal suggests that they have not truly paid a price; rather, they have been elevated to the status of heroes, with their previous engagement in violence securing this position. In contrast, dissidents are not elevated but willingly accept the price of sacrificing their humanity for the community. The community itself is depicted as needing to bear a cost; without their support and sacrifice, a united Ireland cannot be achieved. Thus, the cost of war is framed as worthwhile, as it promises to secure the future. This perspective may help explain the reluctance within the republican community to address the mental health repercussions of the Troubles. To confront the root causes of the mental health crisis is to question the necessity of the armed struggle, which in turn challenges the very foundation of republican identity. Acknowledging this would imply that the republican paramilitary groups, rather than protecting the community, have caused harm, thereby transforming heroes into perpetrators and undermining the basis of power for these groups.

This analysis reveals that within republicanism, there is not only sanctity associated with death but also a sanctity attached to sacrifice and the ability to remain psychologically unscathed by violence. This complicates the established hierarchy within republicanism. Both mainstream and dissident visual cultures highlight a hierarchy and competition within republicanism, as well as within the factions of mainstream and dissident groups. These findings challenge Rolston's and McKeown's discussions of republican homosociality, which argue that republican prisoners prioritized male fraternity and solidarity over competition, creating a communal experience that helped them endure incarceration (Rolston and McKeown, 2017). However, Rolston and McKeown do not address what happened *after* incarceration and the impact of the communal experience on the prisoners' bodies and psyches. The mental health crisis in Northern Ireland, a consequence of the Troubles, underscores the fact that many in the Northern Irish population have either lacked access to or rejected psychological and psychiatric help (Bunting et al., 2013). Indicators of poor mental health, such as lack of initiative and agency in individuals' everyday lives, when considered in relation to these murals' narratives, suggest that a true republican, and by extension a true Irish person, is one who acts. The emphasis on agency and action implies that those who are not active are perceived as lesser. As demonstrated in this analysis, the ability to cope with the repercussions of violence solidifies an individual's place within their identity and collective memory. This illustrates how visual representations play a crucial role in establishing and affirming power relations within identities and collective memories.

This article has demonstrated how a visual culture imbued with meanings of cultural violence influences the understanding of physical and psychological ailments. Collective memory serves as a framework within which personal experiences are contextualized, and the community situates their experiences of war within the visual culture produced and represented by murals. Although there are opposing voices to these messages, the totality of these narratives must be considered when discussing how Northern Ireland as a society can move forward. Especially, regarding how the violence of the Troubles has inflicted a societal wound, one that is continually kept open by ongoing cultural violence. There is a lack of resources to heal this wound, and instead, a persistent visual culture laden with meanings of cultural violence reinforces the notion that the violence was justified. However, this visual culture excludes those who feel otherwise. Therefore, this article has highlighted the complex relationships between collective memory, identities, cultural violence, and bodily experiences. It has also shown the importance of understanding bodily experiences and the consequences when they are not adequately addressed.

English (2007, p. 371) has described the Provisional IRA as both the symptom and the cause of the outbreak of the Troubles, as they were protectors and defenders of their community as they were in the community. This has continued into peace process Northern Ireland regarding mental health problems. In examining the dissident and mainstream republican displays of sacrifice, this article has demonstrated that Sinn Féin faces significant challenges in addressing the mental health issues arising from the Troubles without undermining its own political position. These psychological issues cannot be solely attributed to British security forces; republican paramilitary groups have also inflicted, and continue to inflict, violence upon their own communities. In this way, the study highlights how the “see-saw relationship” identified by Knox extends beyond street-level interactions. By not acknowledging the harmful ways the republican violence has impacted onto their communities means that the leading republicans are still subjecting their communities to violence. This dynamic reaches into Stormont and is maintained in part through murals. The cultural violence embodied in these murals is deeply rooted in collective memory, communicated through practices and expressions beyond the murals themselves. As such, murals offer a lens through which this relationship can be analyzed, and further research could explore how politicians discuss the theme of sacrifice for the unification of Ireland to uncover how cultural violence manifests in other forms.

To control the past is to control the present, but this article has shown that to control the past, one must align with it. Moreover, as collective memory is linked to change and constitutes a form of negotiation among group members, there is an ongoing negotiation of how the present is understood through the lens of the past. This suggests that there is potential to reinterpret the necessity of sacrifice and its place within collective memory. As there is a sizeable part of the population that is suffering from the pain of the Troubles, there might be those who want a different interpretation of the Troubles. The bodily experiences might create a new interpretation of the past. However, such a reinterpretation would require a new understanding of the Troubles, in which Irish-Catholic victimhood is also re-evaluated. In this reinterpretation, the critical question that arises is whether the troubling cost was worth it, and if not, what alternative paths could be pursued.

## Data Availability

The original contributions presented in the study are included in the article/supplementary material, further inquiries can be directed to the corresponding author/s.

## Footnotes

^1^In republican history, resistance against the British government is conceptualized as an ongoing and continuous rebellion. The republican armed struggle is thus perceived as a continuous historical process, composed of distinct yet interconnected events. For instance, the 1981 Hunger Strike, while occurring during the Troubles, is commemorated as a distinct and significant event within the broader republican narrative.

^2^The tradition of murals stretches back to the Partition of Ireland in 1912, in which the unionist government of Ireland utilized murals to establish, enforce and maintain the Protestant Supremacy. This form of the tradition died in the 1950s. The current form of the tradition was born out of the Hunger Strike of 1981 in which it took its rapid form today. Loyalists started to paint mural in this manner in 1984/1985 (Larsson, 2021).

^3^Republicans call the Peace Agreement the “Good Friday Agreement”, whereas loyalists refer to it as the “Belfast Agreement”. I have chosen to label it the Agreement.

^4^The first and second hunger strike was about the British removing the special category status of the republican and loyalist prisoners. Before 1981 they were treated as Prisoners of War: they were exempt from wearing prison uniform, doing prison work and they were allowed to keep their organizational unit in the prison.

^5^Peace babies refer to the generations born after 1998.
